# Quasi-Three-Dimensional Cyclotriphosphazene-Based Covalent Organic Framework Nanosheet for Efficient Oxygen Reduction

**DOI:** 10.1007/s40820-023-01111-8

**Published:** 2023-06-29

**Authors:** Jianhong Chang, Cuiyan Li, Xiaoxia Wang, Daohao Li, Jie Zhang, Xiaoming Yu, Hui Li, Xiangdong Yao, Valentin Valtchev, Shilun Qiu, Qianrong Fang

**Affiliations:** 1 State Key Laboratory of Inorganic Synthesis and Preparative Chemistry, Jilin University, Changchun, 130012 People’s Republic of China; 2 State Key Laboratory of Bio-Fibers and Eco-Textiles, College of Materials Science and Engineering, Qingdao University, Qingdao, 266071 People’s Republic of China; 3 Qingdao Institute of Bioenergy and Bioprocess Technology, Chinese Academy of Sciences, 189 Song Ling Rd, Qingdao, 266101 Shandong People’s Republic of China; 4 Laboratoire Catalyse et Spectrochimie, CNRS, ENSICAEN, UNICAEN, Normandie Univ, 6 Marechal Juin, 14050 Caen, France

**Keywords:** Covalent organic framework, Quasi-three-dimensional structures, Oxygen reduction reaction, Metal-free electrocatalyst

## Abstract

Metal-free carbon-based materials are considered as promising oxygen reduction reaction (ORR) electrocatalysts for clean energy conversion, and their highly dense and exposed carbon active sites are crucial for efficient ORR. In this work, two unique quasi-three-dimensional cyclotriphosphazene-based covalent organic frameworks (Q3CTP-COFs) and their nanosheets were successfully synthesized and applied as ORR electrocatalysts. The abundant electrophilic structure in Q3CTP-COFs induces a high density of carbon active sites, and the unique bilayer stacking of [6 + 3] imine-linked backbone facilitates the exposure of active carbon sites and accelerates mass diffusion during ORR. In particular, bulk Q3CTP-COFs can be easily exfoliated into thin COF nanosheets (NSs) due to the weak interlayer π–π interactions. Q3CTP-COF NSs exhibit highly efficient ORR catalytic activity (half-wave potential of 0.72 V vs. RHE in alkaline electrolyte), which is one of the best COF-based ORR electrocatalysts reported so far. Furthermore, Q3CTP-COF NSs can serve as a promising cathode for Zn-air batteries (delivered power density of 156 mW cm^–2^ at 300 mA cm^–2^). This judicious design and accurate synthesis of such COFs with highly dense and exposed active sites and their nanosheets will promote the development of metal-free carbon-based electrocatalysts.

**Supplementary Information:**

The online version contains supplementary material available at 10.1007/s40820-023-01111-8.

## Highlights

- JUC-610-nanosheet exhibits highly efficient oxygen reduction reaction (ORR) catalytic activity in alkaline electrolyte with half-wave potential of 0.72 V versus reversible hydrogen electrode, which is one of the best covalent organic frameworks (COF)-based ORR electrocatalysts reported so far.
- It has been confirmed by experiments and density functional theory calculations that the abundant electrophilic structure in Q3CTP-COFs induces a highly density of carbon active sites, and the unique bilayer stacking facilitates the exposure of active carbon sites and accelerates the mass diffusion during ORR.
- JUC-610-nanosheet can also serve as a promising cathode for Zn-air batteries (power density of 156 mW cm^–2^ at 300 mA cm^–2^), which promotes the development of metal-free carbon-based electrocatalysts.

**Supplementary Information:**

The online version contains supplementary material available at 10.1007/s40820-023-01111-8.

## Graphical abstract

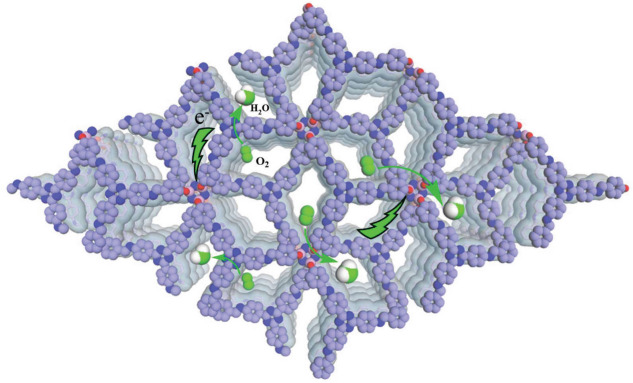

## Introduction

Oxygen reduction reaction (ORR) plays a significant role in clean and sustainable energy conversion, such as metal-air batteries and fuel cells [1–4]. Pt-based materials are currently considered as most efficient electrocatalysts for ORR. However, their high-cost, scarcity, and instability in operation conditions restrict the future large-scale applications of these energy conversion devices [5–7]. In the past decade, metal-free materials (MFMs), such as porous carbon and porous organic polymers, have exerted a great effect on the design of low-cost and efficient electrocatalysts for ORR [8–15]. Notably, covalent organic frameworks (COFs) are an emerging class of ORR electrocatalysts due to their high surface areas, tunable porous structures, structural tunability, and well-defined building units [16–20]. Reasonably designing and synthesizing COF materials with chemically adjustable specific blocks can facilitate the development of MFM-based ORR electrocatalysts [21–23]. During ORR electrocatalysis process, efficient active sites and fast kinetic mass diffusion of electrocatalysts are indispensable ingredients. Expectantly, it has been confirmed through both experiment and theory that the electronic redistribution of carbon (C) atoms in MFMs can optimize the adsorption and dissociation behaviors for reactant (O_2_) and intermediates (OOH*, O*, and OH*) [24–26], inducing ORR catalytic activity. For example, Dai et al. [27] and Nakamura et al. [28] demonstrated that the C atoms with Lewis basicity (positive charge density) were the efficient ORR active sites. Thus, constructing electrophilic structures in COFs can induce positively charged carbon active sites. Furthermore, optimal pore structures to expose more active sites and excellent conductivity of electrocatalysts can accelerate mass diffusion and electron transport to facilitate the ORR catalytic process [29]. Hence, controllably and precisely synthesizing COFs with highly dense and exposed carbon active sites from the perspective of customizing structures is of great potential to develop highly active metal-free ORR electrocatalysts [30].

Among COF materials, three-dimensional (3D) COFs exhibit hierarchical pore structures to expose numerous open sites [31] profitably, but the structural instability and poor conductivity limit their practical applications as electrocatalysis. Two-dimensional (2D) COFs with excellent interlayer regulation have significant advantages in stable structures and electron transport properties [32, 33] but are restricted by mass diffusion due to their 2D stacking [34, 35]. In view of this situation, it may be possible to construct efficient 2D COF electrocatalysts with quasi-3D (Q-3D) structures and channel features through clever structural design. However, the emergence of structural units centered on the Q-3D cyclotriphosphazene structure makes our hypothesis plausible. The distinctive structure of Q-3D COF makes its 2D planar structure regularly distort, thus creating a new vertical multi-channel to enhance the pore permeability and the mass diffusion [36]. Moreover, the weak interlayered π–π interactions of Q-3D COFs can be easily exfoliated into nanosheets (NSs) [37]. Due to large number of active sites, faster ion diffusion, and high conductivity of thin layers, COF NSs was expected to further improve the performance of electrocatalysts [38–41]. Therefore, the active carbon sites and mass diffusion requirements of ORR electrocatalysts initiate us to judiciously design and accurately synthesize the novel Q-3D COF NSs with highly exposed carbon active sites for efficient ORR.

Herein, we report the design and synthesis of two unique Q-3D cyclotriphosphazene-based COFs (Q3CTP-COFs, namely JUC-610 and JUC-611, JUC = Jilin University China) with 2D stacking structures and their exfoliated nanosheet (JUC-610-CON) as efficient ORR electrocatalysts. The electrophilic structures of N–P–O blocks in 6-node hexa(4-formyl-phenoxy) cyclotriphosphazene (CTP-6-CHO) and pyridinic-N in 2,4,6-Tris(4-aminophenyl) triazine (TAPT) induce the highly dense C (positively charged) ORR active sites of JUC-610. The novel [6 + 3] imine-linked frameworks [42, 43] exhibit unique bilayer stacking to expose more active sites and promote mass diffusion during ORR. Furthermore, the weak π–π stacking effect between layers of Q3CTP-COFs makes possible to exfoliate ultra-thin nanosheets (~ 4 nm) easily, which can act as an ORR electrocatalyst with half-wave potential of 0.72 V versus RHE in alkaline electrolyte and the cathode for Zn-air batteries (ZABs) with the power density of 156 mW cm^–2^ at 300 mA cm^–2^, which surpasses those of almost all reported COF materials [20–23]. This work represents a newly synthesized COF architecture for efficient ORR and demonstrates its promising potential in metal-air batteries.

## Experiment and Characterization

### Experiment and Structural Determination of Q3CTP-COFs

**Scheme 1:**
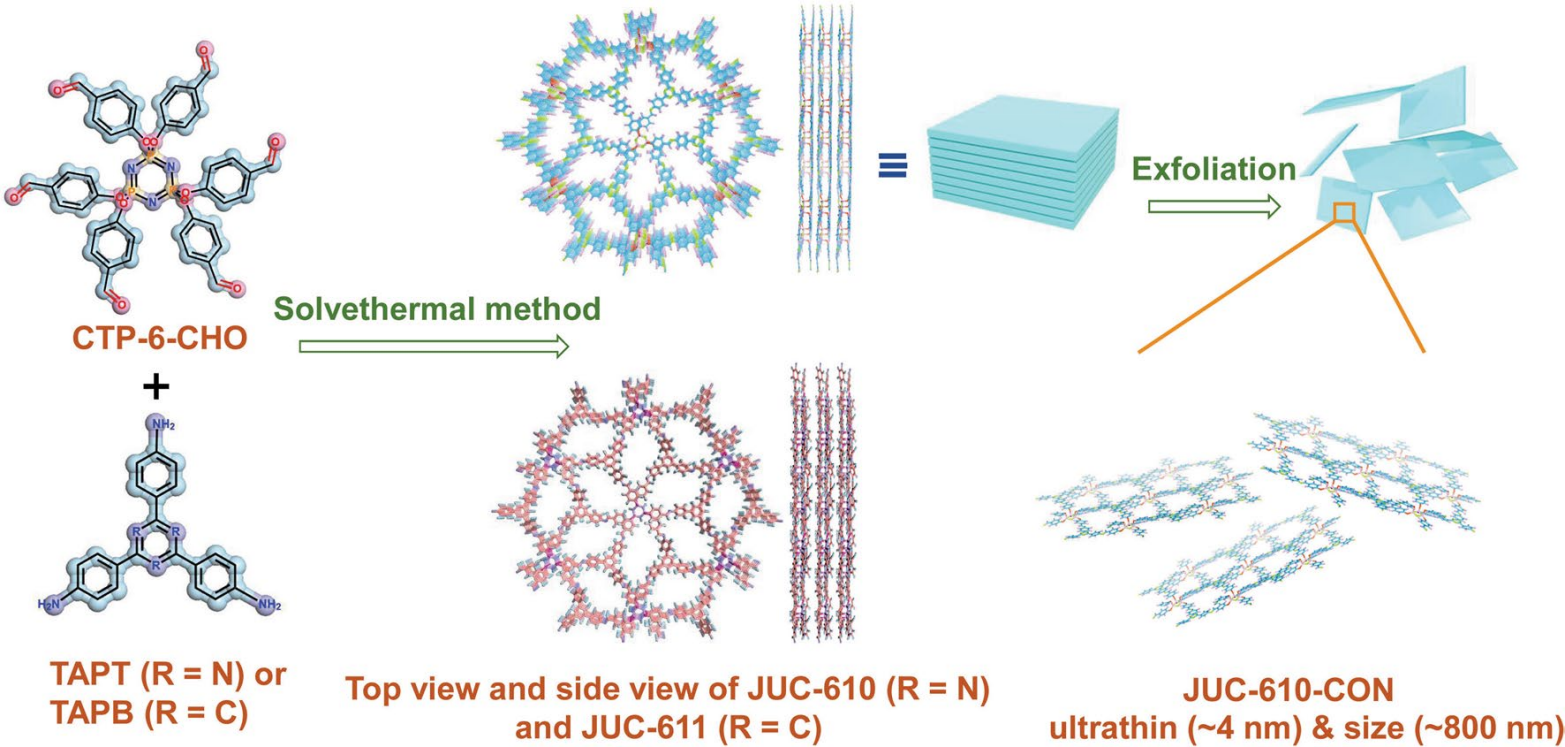
Schematic illustration of constructing JUC-610, JUC-611 (in the mixed solution of o-DCB/n-BuOH under 120 °C for 72 h with the yields of 75% and 72% respectively), and JUC-610-CON

**Fig. 1:**
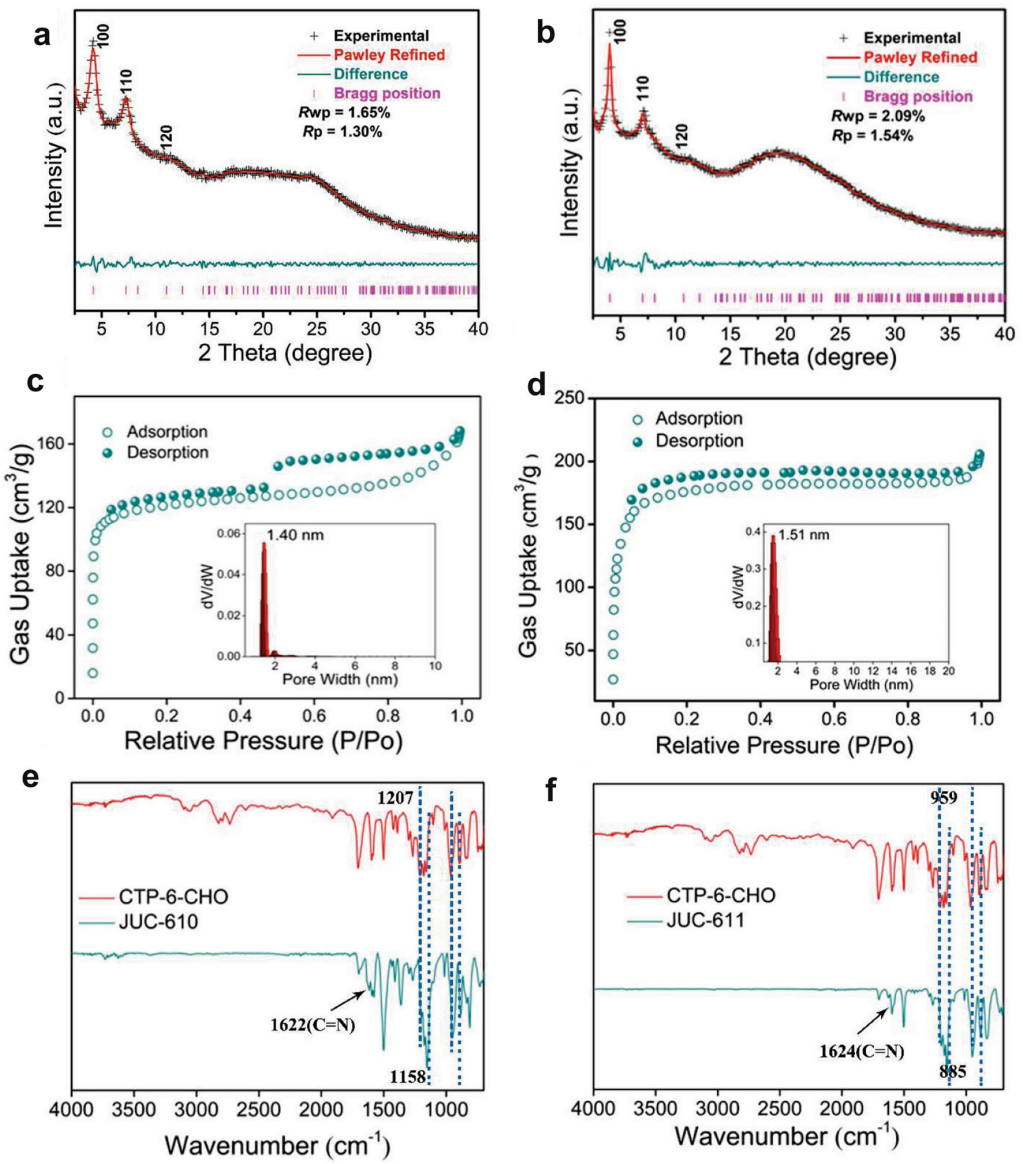
**a, b** Powder XRD patterns of JUC-610 and JUC-611. **c, d** N_2_ adsorption–desorption isotherms at 77 K and pore-size distribution (inset) based on the NLDFT model to the adsorption data for JUC-610 and JUC-611. **e, f** FT-IR spectra of JUC-610 and JUC-611

As shown in Scheme 1 and experimental procedures, Q3CTP-COFs were synthesized by condensing CTP-6-CHO with TAPT or 2,4,6-Tris(4-aminophenyl)benzene (TAPB) in the mixed solution of *o*-DCB/*n*-BuOH under 120 °C for 72 h with the yields of 75% and 72%, respectively. The powder X-ray diffraction (PXRD) patterns suggested that the crystal structures of Q3CTP-COFs well matched with simulated by using the Materials Studio software package (version 7.0) [44] based on the bilayer ***hcb*** net [45] (Fig. 1a, b). After geometrical energy minimization by using the force field to optimize the geometry of the molecular building blocks, the unit cell parameters (*a* = *b* = 24.6620 Å, *c* = 5.9759 Å and *α* = *β* = 90°, *γ* = 120° for JUC-610; *a* = *b* = 25.6008 Å, *c* = 6.4398 Å and *α* = *β* = 90°, *γ* = 120° for JUC-611, respectively) were obtained. Based on *P3* space group (No. 143), Bragg peaks at 2*θ* = 4.14°, 7.18°, and 10.95° for JUC-610 were correspond to the (100), (110) and (120) planes, and peaks at 2*θ* = 4.02°, 6.97° and 10.65° for JUC-611 were correspond to the (100), (110), and (120) planes, respectively. The refinement results yielded unit cell parameters nearly equivalent to the predictions (*R*_*wp*_ = 1.65% and *R*_*p*_ = 1.30% for JUC-610; *R*_*wp*_ = 2.09% and *R*_*p*_ = 1.54% for JUC-611). A comparison between experimental and calculated PXRD curves (AA and AB stacking models) revealed that both COFs crystallized in AA stacking mode (Figs. S1–S2 and Tables S1–S4).

### Characterization of Q3CTP-COFs

**Fig. 2:**
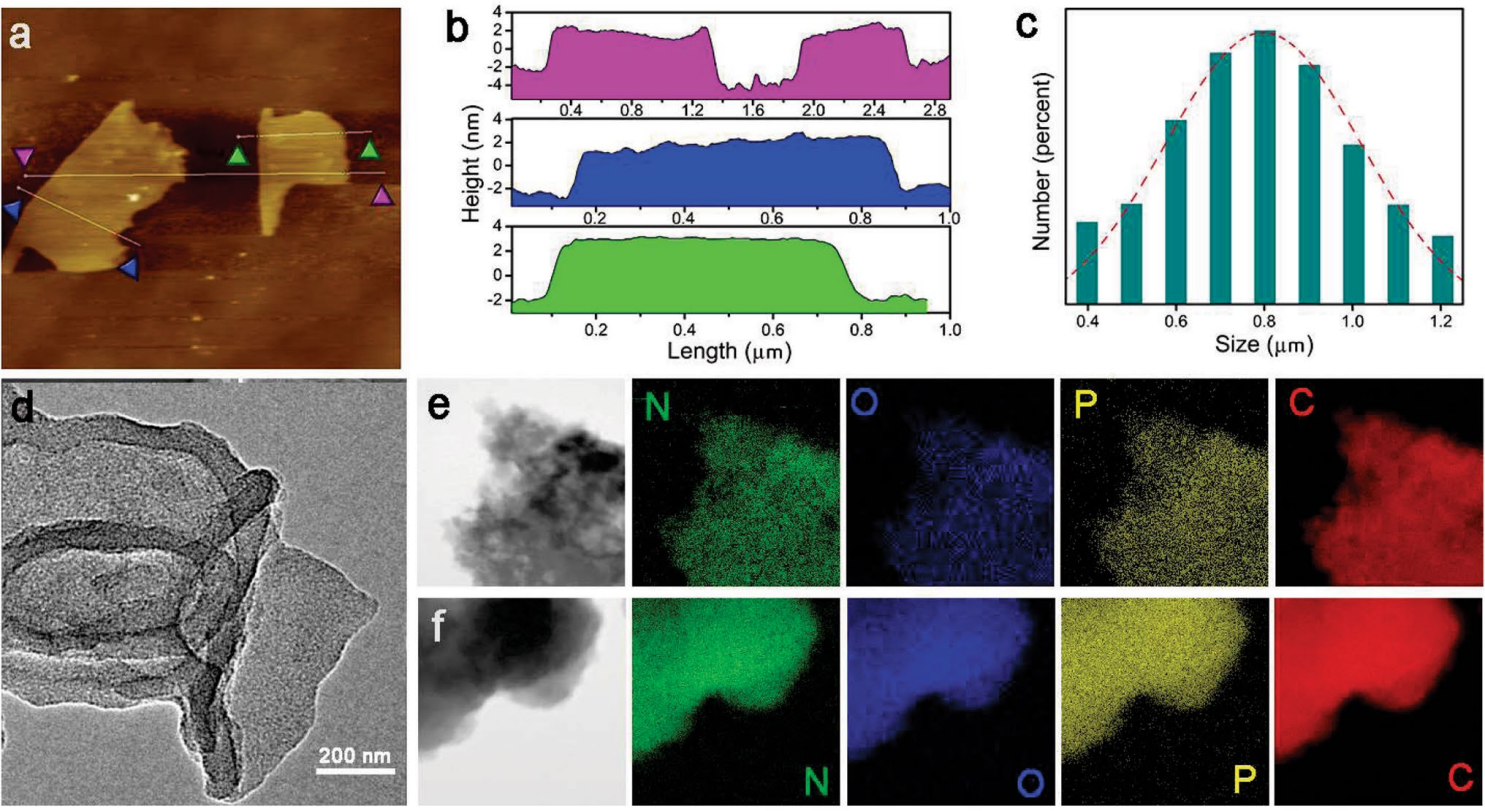
**a** AFM image of JUC-610-CON, and **b** the corresponding height curves for the selective areas in **a**. **c** Lateral size distribution histogram of JUC-610-CON, and Gaussian fit curve is colored in red. **d** TEM image of JUC-610-CON. **e, f** TEM and the related elemental mapping images of carbon, nitrogen, oxygen, and phosphorus for JUC-610 and JUC-611

Nitrogen adsorption–desorption analysis under 77 K of both prepared COFs showed a sharp uptake at a low pressure of *P*/*P*_0_ < 0.05, which is a typical characteristic of microporous material [46–48] (Fig. 1c, d). The pore size distribution was calculated on the basis of non-local density functional theory (NLDFT), resulting in a value of 14.0 Å for JUC-610 and 15.1 Å for JUC-611 respectively, which is in good agreement with those of the proposed models (15.6 Å for JUC-610 and 15.8 Å for JUC-611). The Brunauer–Emmett–Teller (BET) equation revealed their surface areas, 475 m^2^ g^−1^ for JUC-610 and 694 m^2^ g^−1^ for JUC-611 (Figs. S4–S5). The Fourier transform infrared (FT-IR) spectra peak around 1622 cm^−1^ for JUC-610 and 1624 cm^−1^ for JUC-611 indicate the formation of C=N (Figs. 1e, f and S6–S7). Interestingly, the FT-IR spectra of CTP-6-CHO, Q3CTP-COFs showed that the P=N/P–O–Ar bonds on the phosphazene ring had barely noticeable different shifts with the peaks of P=N bond at 1207, 1158, and 885 cm^−1^ and P–O–Ar bond at 959 cm^−1^ (Fig. 1e, f). Furthermore, the solid-state ^13^C cross-polarization magic-angle-spinning (CP/MAS) NMR spectra verified that the presence of carbons from the C=N bonds at 162 ppm for JUC-610 and 161 ppm for JUC-611 (Figs. S8–S9). According to the thermogravimetric analysis (TGA), these prepared COFs showed excellent thermal stability and were thermally stable up to ~ 400 °C under nitrogen (Figs. S10–S11). The morphologies of prepared COFs were observed by scanning electron microscopy (SEM) and transmission electron microscope (TEM), in which Q3CTP-COFs showed isometric crystals (Figs. S12–S13). The powder X-ray diffraction (PXRD) patterns revealed that COFs were stable after immersing in acetone and THF solvents and alkaline aqueous solutions for 24 h (Figs. S14–S16). The FT-IR spectra of COFs showed that the C=N bonds still existed after treatment under 6 M KOH for 24 h (Figs. S17–S19). Interestingly, we elaborately selected CTP-6-CHO with the structure of six cross-side arms outside the central plane as the node module of COFs. The CTP-6-CHO has a unique stereoscopic structure in which the O–P–O plane (*β* plane) is perpendicular to the aromatic ring plane of N_3_P_3_ (*α* plane, see Experimental Procedures) [37, 49]. In addition, the TEM images with elemental mappings verified that C, N, O, and P atoms were homogeneously distributed in Q3CTP-COFs (Fig. 2d-f).

### Experiment and Characterization of JUC-610-CON

In addition, given consisting of unique rhombus pores, large interlayer spacings, and the uniquely flexible structure of CTP-6-CHO units lead to the weakened interlayer conjugation and possess the larger *c* distance (Table S1) of JUC-610, and the bulk JUC-610 can be easily exfoliated into ultrathin NSs (denoted as JUC-610-CON) in large quantities (see Supporting Information) only 3 h by using sonication method. The ultrathin nature of JUC-610-CON was uncovered by TEM and AFM technologies. Tyndall effect was observed when a green laser went through the solution of JUC-610-CON (Fig. S20), confirming its colloidal nature. The atomic force microscopy (AFM) image revealed that the thickness of the obtained JUC-610-CON was ~ 4 nm, corresponding to ~ 8 layers and a flake size of ~ 800 nm (Fig. 2a–c and Table S7). Meanwhile, JUC-610-CON exhibited thin nanosheets by TEM and SEM (Figs. 2d and S21). To determine the crystal structure of JUC-610-CON, XRD and nitrogen adsorption–desorption analyses were performed. The results indicate the crystallinity and porosity of JUC-610-CON are identical to that of the bulk JUC-610, but the intensities of the first XRD peak (100) and N_2_ adsorption isotherm are decreased (Figs. S22–S23).

## Results and Discussion

### ORR Catalytic Activity

**Fig. 3:**
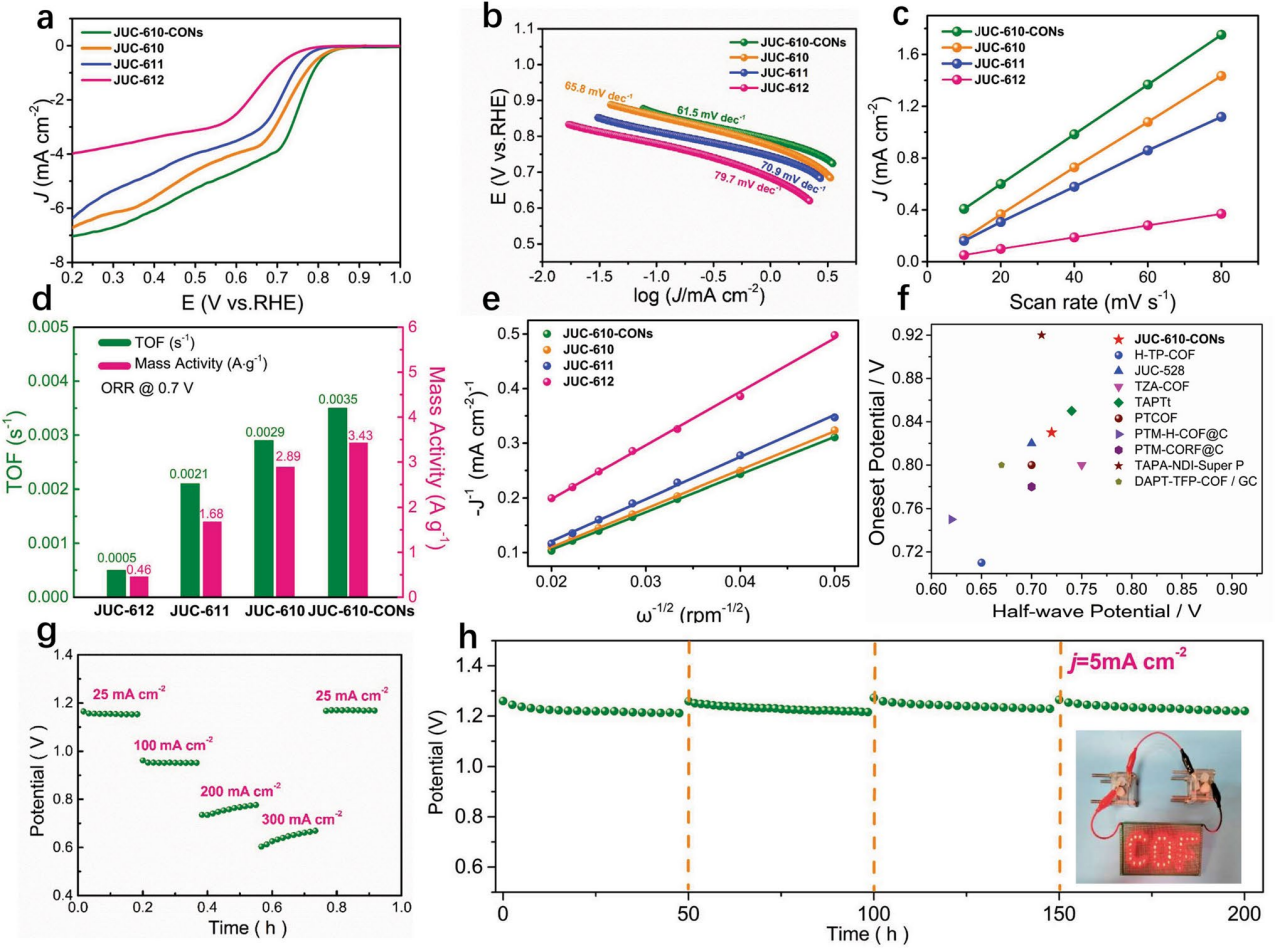
**a** LSV curves of JUC-610-CON, JUC-610, JUC-611, and JUC-612 at 1600 rpm in O_2_-saturated 0.1 M KOH electrolyte. **b** Comparison of Tafel plots, **c** estimated electrochemical bilayer capacitance (*C*_dl_). **d** TOF and mass activity, and **e** *K–L* plots for JUC-610-CON, JUC-610, JUC-611, and JUC-612. **f** Plot of onset potential against half-wave potential in metal-free organic porous materials without pyrolysis of ORR reactions. **g** Discharge curves of JUC-610-CON-based ZAB at different current densities (25, 100, 200, and 300 mA cm^−2^). **h** The stability of JUC-610-CON-based ZAB at 5 mA cm^−2^ in ambient air conditions, and photographs of red “COF” LED panel powered by two ZABs in series (inset)

**Scheme 2:**
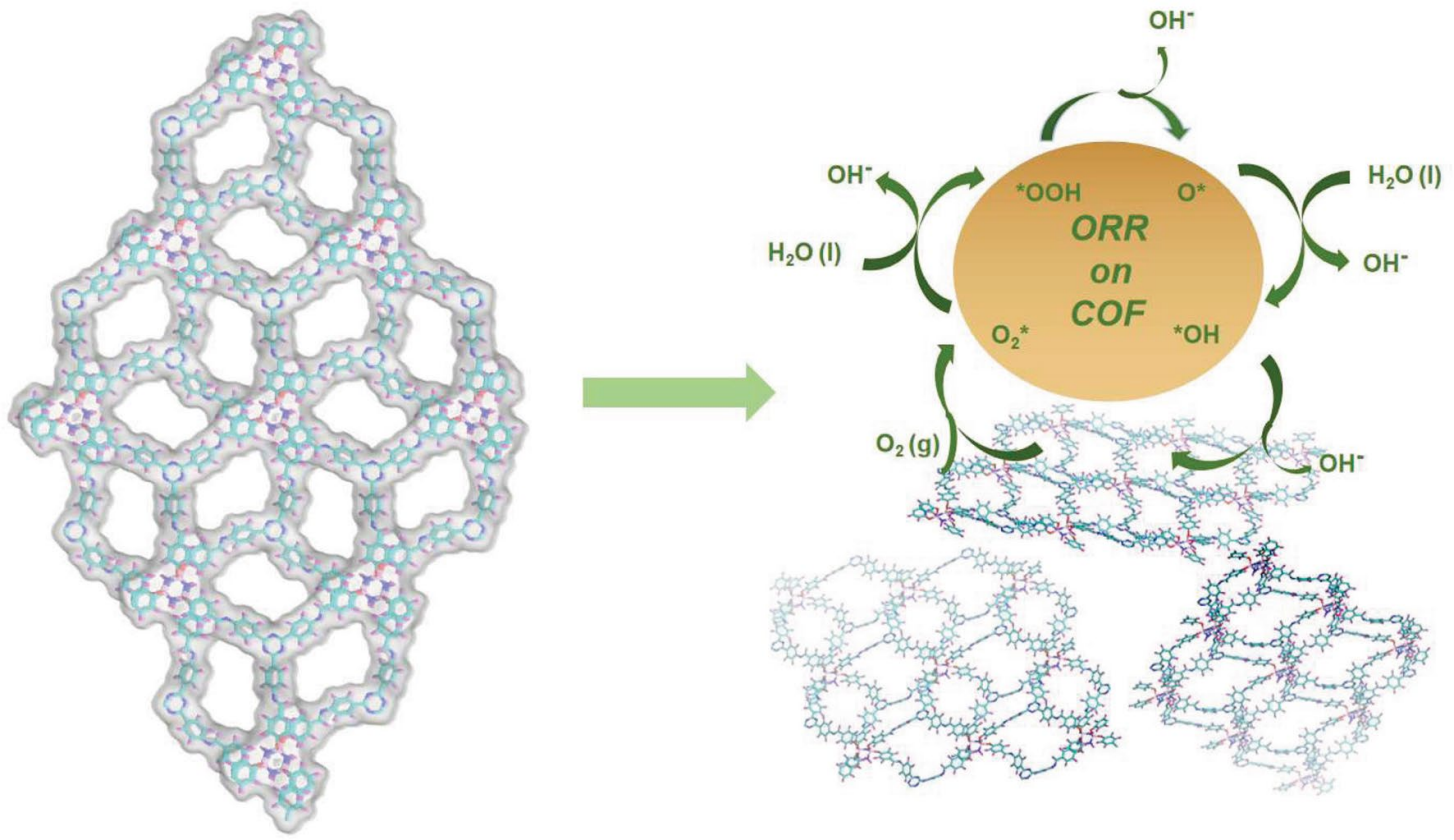
Proposed ORR processes on JUC-610-CON

To survey the ORR catalytic activity of prepared COFs and their nanosheets, the rotating disk electrode (RDE) experiments were performed in O_2_-saturated 0.1 M KOH electrolyte. In addition, JUC-612 was also synthesized by condensing 1,3,5-tri(1,3-Diformyl biphenyl)benzene (TBPB-6-CHO) and TAPT as a control sample for ORR performance (Figs. S24–S31). According to the linear sweep voltammetry (LSV) curves, JUC-610-CON and Q3CTP-COFs exhibited higher ORR performance than that of JUC-612, revealing that the existence of the Q-3D structures and abundant heteroatoms significantly induces positively charged carbon active sites and improves the ORR active sites (Figs. 3a, S32–S36, and Scheme 2). The ORR catalytic activity of JUC-610 was superior to that of JUC-611, which indicates that N atoms in TAPT blocks also play an important role in improving the ORR performance. In addition, the half-wave potential of JUC-610-CON and the bulk JUC-610 were 0.72 and 0.69 V versus RHE, respectively (Fig. S37), suggesting that ultrathin nanosheet structure exposes more active sites and facilitates mass diffusion during ORR process, which can be comparable to previously reported COF materials and most of metal-based ORR electrocatalysts (Fig. 3f and Tables S8–S9). The Tafel slope of JUC-610-CON (61.95 mV dec^−1^) is lower than those of JUC-610 (65.8 mV dec^−1^), JUC-611 (70.9 mV dec^−1^) and JUC-612 (79.7 mV dec^−1^), indicating the superior ORR kinetics of JUC-610-CON (Fig. 3b). The electrochemically active surface areas (ECSA) of prepared samples were conducted by electrochemical double-layer capacitance (*C*_dl_) (Figs. S38–S41). The *C*_dl_ of JUC-610-CON (19.2 mF cm^−2^) is larger than those of JUC-610 (17.8 mF cm^−2^), JUC-611 (7.6 mF cm^−2^), and JUC-612 (7.6 mF cm^−2^, Fig. 3c). To further explore the intrinsic activity of the prepared COFs, the turnover frequency (TOF) was carried out at 0.7 V versus RHE, indicating that C atoms in adjacent N–O–P atoms are active sites, and the highly dense carbon active sites accelerate mass diffusion during ORR process. As shown in Fig. 3d, the TOF value of JUC-610-CON is 0.0035 s^−1^, which has higher active site utilization efficiency than Q3CTP-COFs and JUC-612. The mass activity of JUC-610-CON (3.43 A g^−1^) is also larger than those of JUC-610 (2.89 A g^−1^), JUC-611 (1.68 A g^−1^), and JUC-612 (0.46 A g^−1^). These results thus reveal that the JUC-610-CON exhibits the most efficient ORR catalytic performance due to ultrathin nanosheet structure and the highly dense carbon active sites. Moreover, all the electron transfer numbers (n) of JUC-610-CON, JUC-610, JUC-611, and JUC-612 derived from Koutecky–Levich (K–L) plots at 0.2 V versus RHE (Fig. 3e) were closed to 4 (3.82, 3.43, 3.42, and 3.67 respectively).

Subsequently, a ZAB was assembled using the JUC-610-CON as the air cathode due to its excellent ORR activity in alkaline solutions. The current density of JUC-610-CON-based ZAB was about 87.2 mA cm^−2^ at 1.0 V, and the maximum peak power density at 0.60 V was about 0.15 W cm^−2^ (Fig. S42). In addition, the JUC-610-CON-based ZAB also exhibited potential plateaus of 1.16, 0.95, 0.76, and 0.64 V at discharge current densities of 25, 100, 200, and 300 mA cm^−2^, respectively (Fig. 3g). Such ZAB could operate for 200 h with negligible voltage loss by regular replacement of electrolyte (6 M KOH) and zinc plate (Fig. 3h). Two JUC-610-CON-based ZABs in series were able to light a 2 V rated “COF” LED (Fig. 3h inset), which indicates this material is very promising as electrode material in metal-air batteries.

### DFT Calculations

**Fig. 4:**
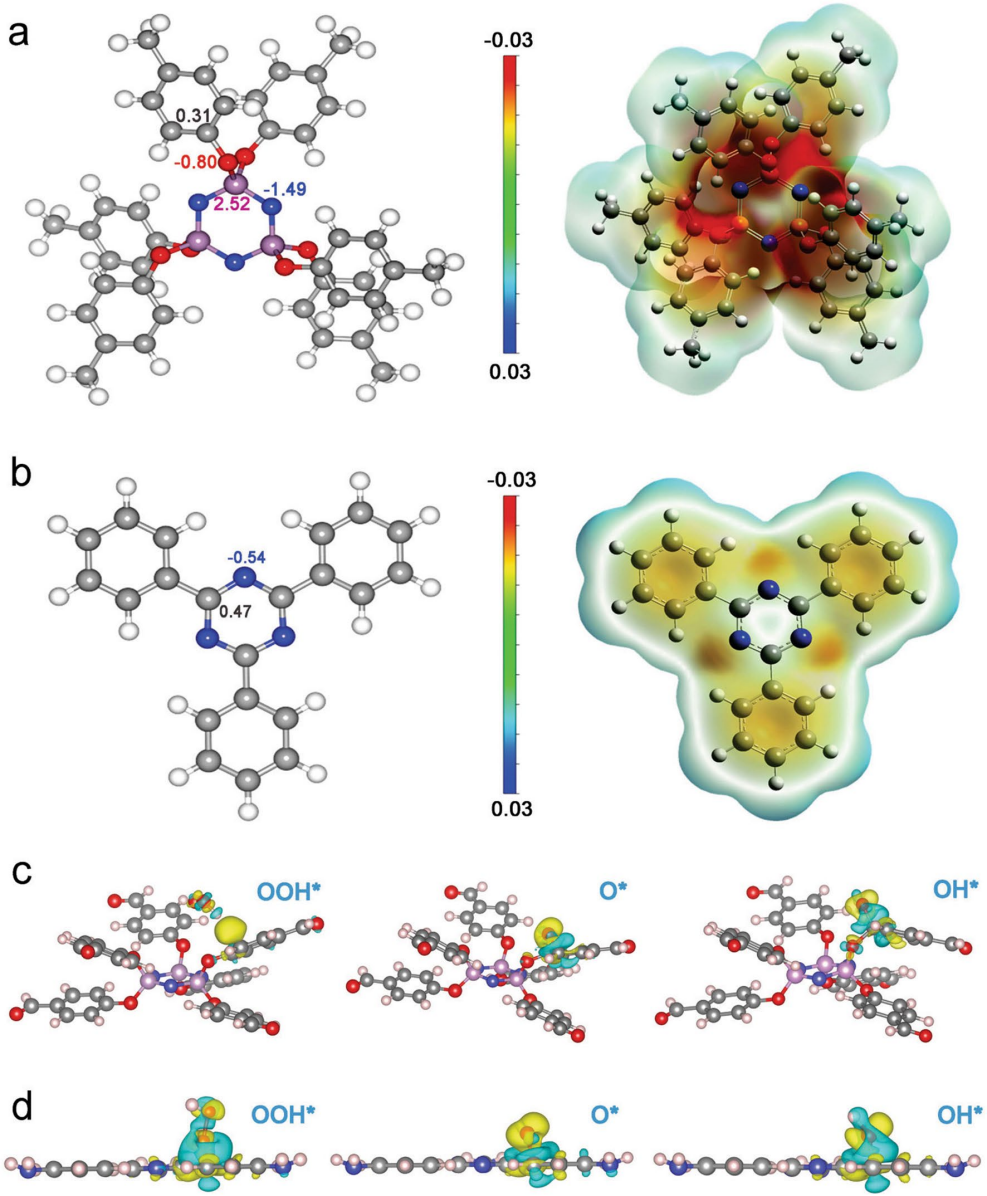
**a, b** NPA charges and the corresponding surface electrostatic potential maps of CTP-6-CHO and TAPT calculated by DFT. **c, d** Side view of the 3D charge densities for the three ORR reaction steps of C site for CTP-6-CHO and TAPT. Gray, purple, red, blue and light pink balls represent C, P, O, N, and H atoms, respectively

To reveal the location of carbon active sites for ORR in our samples, the DFT calculations were performed (Fig. 4). All calculations were carried out with the Gaussian 09 package and Vienna Ab-initio Simulation Package (VASP). The natural population analysis (NPA) was performed on the theoretical level of B3LYP/6-311G (d, p) using the NBO program. The electrostatic potential was considered in Gaussian 09 to describe the charge distribution of two chemical systems [50, 51]. The average electrostatic potential of O atoms in CTP-6-CHO and N atoms in TAPT were smaller than those of their adjacent C atoms, indicating the relative electrophilicity of the N and O atoms. The NPA charge results illustrated that both C atoms adjacent to the electrophilic O atom in CTP-6-CHO and the electrophilic N atom in TAPT showed positive charge, which are 0.31 and 0.47, respectively (Fig. 4a, b). Moreover, for these active carbon sites in CTP-6-CHO and TAPT, the 3D charge densities of three reactions of ORR exhibited obvious redistribution of electrons between the intermediate and substrate structure, verifying the favorable adsorption of positively charged C atoms to the ORR intermediate.

## Conclusions

In summary, we have precisely and controllably synthesized the unique Q3CTP-COFs and their nanosheets using CTP-based blocks, which can act as efficient ORR electrocatalysts for Zn-air batteries. It was confirmed that the electrophilic structures in such Q3CTP-COFs induced abundant positively charged carbon ORR active sites to facilitate O_2_ adsorption and reduction, which has been supported by DFT calculations. The unique bilayer stacking structures of Q3CTP-COFs promote the exposure of active carbon sites to accelerate ORR process and the mass (ions, O_2_ and intermediate) diffusion efficiency during ORR. Furthermore, Q3CTP-COFs could be easily exploited into nanosheets, which improves their ORR catalytic activity (half-wave potential of 0.72 V vs. RHE in alkaline electrolyte) and can be applied for promising cathodes for Zn-air batteries (delivered power density of 156 mW cm^–2^ at 300 mA cm^–2^). Thus, this work provides a new way to fabricate metal-free ORR electrocatalysts with atomically definite carbon active sites and promotes their extensive application in clean energy conversion devices.

### Supplementary Information

Below is the link to the electronic supplementary material.

Supplementary file1 (PDF 3290 KB)

## Abbreviations

ORR: Oxygen reduction reaction
COFs: Covalent organic frameworks
Q-3D: Quasi-three-dimensional
CTP: Cyclotriphosphazene
NSs: Nanosheets
MFMs: Metal-free materials
3D: Three-dimensional
2D: Two-dimensional
JUC: Jilin University China
LSV: Linear sweep voltammetry
ECSA: Electrochemically active surface areas
ZABs: Zn-air batteries
C_dl_: Double-layer capacitance
TOF: Turnover frequency
K–L: Koutecky–Levich

## References

[1] Kumar A, Zhang Y, Liu W, Sun X (2020). The chemistry, recent advancements and activity descriptors for macrocycles based electrocatalysts in oxygen reduction reaction. Coord. Chem. Rev..

[2] Kumar A, Vashistha VK, Das DK (2021). Recent development on metal phthalocyanines based materials for energy conversion and storage applications. Coord. Chem. Rev..

[3] Wang Y, Kumar A, Ma M, Jia Y, Wang Y (2020). Hierarchical peony-like FeCo-NC with conductive network and highly active sites as efficient electrocatalyst for rechargeable Zn-air battery. Nano Res..

[4] Li C, Selvam NCS, Fang J (2023). Shape-controlled synthesis of platinum-based nanocrystals and their electrocatalytic applications in fuel cells. Nano-Micro Lett..

[5] Zhang C, Shen X, Pan Y, Peng Z (2017). A review of Pt-based electrocatalysts for oxygen reduction reaction. Front. Energy.

[6] Cruz-Martinez H, Rojas-Chavez H, Matadamas-Ortiz PT, Ortiz-Herrera JC, Lopez-Chavez E (2021). Current progress of Pt-based ORR electrocatalysts for PEMFCs: an integrated view combining theory and experiment. Mater. Today Phys..

[7] Wang C, Zhang Q, Yan B, You B, Zheng J (2023). Facet engineering of advanced electrocatalysts toward hydrogen/oxygen evolution reactions. Nano-Micro Lett..

[8] Jia Y, Zhang L, Zhuang L, Liu H, Yan X (2019). Identification of active sites for acidic oxygen reduction on carbon catalysts with and without nitrogen doping. Nat. Catal..

[9] Xu Q, Tang Y, Zhang X, Oshima Y, Chen Q (2018). Template conversion of covalent organic frameworks into 2d conducting nanocarbons for catalyzing oxygen reduction reaction. Adv. Mater..

[10] Wu J, Yang Z, Li X, Sun Q, Jin C (2013). Phosphorus-doped porous carbons as efficient electrocatalysts for oxygen reduction. J. Mater. Chem. A.

[11] Li D, Jia Y, Chang G, Chen J, Liu H (2018). A defect-driven metal-free electrocatalyst for oxygen reduction in acidic electrolyte. Chem.

[12] Jia Y, Jiang K, Wang H, Yao X (2019). The role of defect sites in nanomaterials for electrocatalytic energy conversion. Chem.

[13] Zhang J, Chen G, Muellen K, Feng X (2018). Carbon-rich nanomaterials: fascinating hydrogen and oxygen electrocatalysts. Adv. Mater..

[14] Song Y, Ruan P, Mao C, Chang Y, Wang L (2022). Metal–organic frameworks functionalized separators for robust aqueous zinc-ion batteries. Nano-Micro Lett..

[15] Xue Y, Guo Y, Zhang Q, Xie Z, Wei J (2022). MOF-derived Co and Fe species loaded on N-doped carbon networks as efficient oxygen electrocatalysts for Zn-air batteries. Nano-Micro Lett..

[16] Geng K, He T, Liu R, Dalapati S, Tan KT (2020). Covalent organic frameworks: design, synthesis, and functions. Chem. Rev..

[17] Feng X, Ding X, Jiang D (2012). Covalent organic frameworks. Chem. Soc. Rev..

[18] Guan X, Chen F, Fang Q, Qiu S (2020). Design and applications of three dimensional covalent organic frameworks. Chem. Soc. Rev..

[19] Yusran Y, Guan X, Li H, Fang Q, Qiu S (2020). Postsynthetic functionalization of covalent organic frameworks. National Sci. Rev..

[20] Li D, Li C, Zhang L, Li H, Zhu L (2020). Metal-free thiophene-sulfur covalent organic frameworks: precise and controllable synthesis of catalytic active sites for oxygen reduction. J. Am. Chem. Soc..

[21] Roy S, Mari S, Sai MK, Sarma SC, Sarkar S (2020). Highly efficient bifunctional oxygen reduction/evolution activity of a non-precious nanocomposite derived from a tetrazine-COF. Nanoscale.

[22] Park JH, Lee CH, Ju J-M, Lee J-H, Seol J (2021). Bifunctional covalent organic framework-derived electrocatalysts with modulated p-band centers for rechargeable Zn-Air batteries. Adv. Funct. Mater..

[23] Yue Y, Wang T, Wu X, Yang P, Ma Y (2021). Two-dimensional porphyrin covalent organic frameworks with tunable catalytic active sites for the oxygen reduction reaction. Chem. Commun..

[24] Yang B, Miao J, Hung F, Chen J, Tao HB (2016). Identification of catalytic sites for oxygen reduction and oxygen evolution in N-doped graphene materials: development of highly efficient metal-free bifunctional electrocatalyst. Sci. Adv..

[25] Liang J, Jiao Y, Jaroniec M, Qiao SZ (2012). Sulfur and nitrogen dual-doped mesoporous graphene electrocatalyst for oxygen reduction with synergistically enhanced performance. Angew. Chem. Int. Ed..

[26] Su Y, Yao Z, Zhang F, Wang H, Mics Z (2016). Sulfur-enriched conjugated polymer nanosheet derived sulfur and nitrogen Co-doped porous carbon nanosheets as electrocatalysts for oxygen reduction reaction and zinc-air battery. Adv. Funct. Mater..

[27] Gong K, Du F, Xia Z, Durstock M, Dai L (2009). Nitrogen-doped carbon nanotube arrays with high electrocatalytic activity for oxygen reduction. Science.

[28] Guo D, Shibuya R, Akiba C, Saji S, Kondo T (2016). Active sites of nitrogen-doped carbon materials for oxygen reduction reaction clarified using model catalysts. Science.

[29] Wu Q, Jia Y, Liu Q, Mao X, Guo Q (2022). Ultra-dense carbon defects as highly active sites for oxygen reduction catalysis. Chem.

[30] Li D, Long X, Wu Y, Hou H, Wang X (2022). Hierarchically porous and defective carbon fiber cathode for efficient Zn-air batteries and microbial fuel cells. Adv. Fiber. Mater..

[31] Gui B, Ding H, Cheng Y, Mal A, Wang C (2022). Structural design and determination of 3D covalent organic frameworks. Trends Chem..

[32] Yang C, Tao S, Huang N, Zhang X, Duan J (2020). Heteroatom-doped carbon electrocatalysts derived from nanoporous two-dimensional covalent organic frameworks for oxygen reduction and hydrogen evolution. ACS Appl. Nano Mater..

[33] Wang X-T, Lin X-F, Yu D-S (2022). Metal-containing covalent organic framework: a new type of photo/electrocatalyst. Rare Met..

[34] Liu Y, Ren J, Wang Y, Zhu X, Guan X (2022). A stable luminescent covalent organic framework nanosheet for sensitive molecular recognition. CCS Chem.

[35] Ren X, Li C, Kang W, He L, Na T (2021). Enormously promoted photocatalytic activity by using a near-single layer COFs. CCS Chem..

[36] Guo X, Li Y, Zhang M, Cao K, Tian Y (2020). Colyliform crystalline 2d covalent organic frameworks (COFs) with quasi-3d topologies for rapid I(2)adsorption. Angew. Chem. Int. Ed..

[37] Li X, Xu S, Leng K, Chee SW, Zhao X (2020). Partitioning the interlayer space of covalent organic frameworks by embedding pseudorotaxanes in their backbones. Nat. Chem..

[38] Sasmal HS, Mahato AK, Majumder P, Banerjee R (2022). Landscaping covalent organic framework nanomorphologies. J. Am. Chem. Soc..

[39] Mohata S, Dey K, Bhunia S, Thomas N, Gowd EB (2022). Dual nanomechanics in anisotropic porous covalent organic framework janus-type thin films. J. Am. Chem. Soc..

[40] Mahato AK, Bag S, Sasmal HS, Dey K (2021). Crystallizing sub 10 nm covalent organic framework thin films via interfacial–residual concomitance. J. Am. Chem. Soc..

[41] Dey K, Mohata S, Banerjee R (2021). Covalent organic frameworks and supramolecular nano-synthesis. ACS Nano.

[42] Ding Y, Gao J, Wang Q, Zhang Y, Song G (2021). Construction of covalent organic framework for catalysis: Pd/COF-LZU1 in suzuki–miyaura coupling reaction. J. Am. Chem. Soc..

[43] Uribe-Romo FJ, Hunt JR, Furukawa H, Klock C, O'Keeffe M (2009). A crystalline imine-linked 3-d porous covalent organic framework. J. Am. Chem. Soc..

[44] Materials Studio v.7.0 (Accelrys Inc, 2013). http://www.3dsbiovia.com/products/collaborative-science/biovia-materials-studio

[45] Keeffe MO, Peskov MA, Ramsden SJ, Yaghi OM (2008). The reticular chemistry structure resource (RCSR) database of, and symbols for, crystal nets. Acc. Chem. Res..

[46] Fang Q, Wang J, Gu S, Kaspar RB, Zhuang Z (2015). 3D porous crystalline polyimide covalent organic frameworks for drug delivery. J. Am. Chem. Soc..

[47] Ji C, Su K, Wang W, Chang J (2021). Tunable cage based three-dimensional covalent organic frameworks. CCS Chem..

[48] Liu Y, Wu C, Sun Q, Hu F, Pan Q (2020). Spirobifluorene-based three-dimensional covalent organic frameworks with rigid topological channels as efficient heterogeneous catalyst. CCS Chem..

[49] Geng T-M, Wang F-Q, Fang X-C, Xu H (2021). Dual functional N,O,P containing covalent organic frameworks for adsorbing iodine and fluorescence sensing to p-nitrophenol and iodine. Microporous Mesoporous Mater..

[50] Ditchfield R, Hehre WJ, Pople JA (1971). An extended gaussian-type basis for molecular-orbital studies of organic molecules. J. Chem. Phys..

[51] Hehre WJ, Ditchfield R, Pople JA (1972). Self—consistent molecular orbital methods. XII. Further extensions of gaussian—type basis sets for use in molecular orbital studies of organic molecules. J. Chem. Phys..

