# Supplementary material for: Quasi-Three-Dimensional Cyclotriphosphazene-Based Covalent Organic Framework Nanosheet for Efficient Oxygen Reduction
Source: Nanomicro Lett. 2023 Jun 29;15:159. doi: 10.1007/s40820-023-01111-8 (PMC10310679; doi:10.1007/s40820-023-01111-8)
Supplement: Supplementary file 1 — Supplementary file1 (PDF 3290 KB) [file 40820_2023_1111_MOESM1_ESM.pdf]

Supporting Information for

Quasi-Three-Dimensional Cyclotriphosphazene-Based Covalent Organic Framework Nanosheet for Efficient Oxygen Reduction

Jianhong Chang[1, #], Cuiyan Li[1, #], Xiaoxia Wang[2], Daohao Li[2, *], Jie Zhang[1], Xiaoming Yu[1], Hui Li[1], Xiangdong Yao[1], Valentin Valtchev[3, 4], Shilun Qiu[1], and Qianrong Fang[1, *]

[1]State Key Laboratory of Inorganic Synthesis and Preparative Chemistry, Jilin University, Changchun 130012, P. R. China

[2]State Key Laboratory of Bio-fibers and Eco-textiles, College of Materials Science and Engineering, Qingdao University, Qingdao 266071, P. R. China.

[3]Qingdao Institute of Bioenergy and Bioprocess Technology, Chinese Academy of Sciences 189 Song Ling Rd, Qingdao, Shandong 266101, P. R. China
[4]Normandie Univ, ENSICAEN, UNICAEN, CNRS, Laboratoire Catalyse et Spectrochimie, 6 Marechal Juin, 14050 Caen, France

[#]J. Chang and C. Li contributed equally to this work.

*Correspongding authors. E-mail: qrfang@jlu.edu.cn (Qianrong Fang), lidaohao@qdu.edu.cn (Daohao Li)

S1 Materials

S1.1 General Considerations

All starting materials and solvents, unless otherwise noted, were obtained from J&K scientific LTD. The reagents and purity of the solvents were of 95% and used without further purification. Hexa(4-formyl-phenoxy)cyclotriphosphazene (CTP-6-CHO) [S1], Synthesis of 1,3,5-tri(1,3-Diformyl biphenyl)benzene (TDBPB), and 2,4,6-Tris(4-aminophenyl)-1,3,5-triazine (TAPT) [S2], and (TAPB) [S3] were synthesized using a modified literature method. All products were isolated and handled under nitrogen using either glovebox or Schlenk line techniques.

A Bruker AV-400 NMR spectrometer was applied to record the liquid ^{1}H NMR spectra. Solid-state ^{13}C NMR spectra were recorded on an AVIII 500 MHz solid-state NMR spectrometer. The FTIR spectra (KBr) were obtained using a SHIMADZU IRAffinity-1 Fourier transform infrared spectrophotometer. AFM was conducted on a Multimode 8 scanning probe microscope. Thermogravimetric analysis (TGA) was recorded on a SHIMADZU DTG-60 thermal analyzer under N_2. The operational range of the instrument was from 30 to 600 °C at a heating rate of 10 °C min^{-1} with N_2 flow rate of 30 mL min^{-1}. PXRD data were collected on a PANalytical B.V. Empyrean powder diffractometer using a Cu Kα source (λ = 1.5418 Å) over the range

of 2θ = 2.0−40.0° with a step size of 0.02° and 2 s per step. The sorption isotherm for N_2 was measured by using a Quantachrome Autosorb-IQ analyzer with ultra-high-purity gas (99.999% purity). Before gas adsorption measurements, the as-synthesized COFs (~50.0 mg) were immersed in DMF for 12 h (3 × 5.0 ml) and then acetone for another 36 h (3 × 5.0 ml). The acetone was then extracted under vacuum at 85 °C to afford the samples for sorption analysis. To estimate pore size distributions for COFs, nonlocal density functional theory (NLDFT) was applied to analyze the N_2 isotherm based on the model of N_2@77K on carbon with slit pores and the method of non-negative regularization. For scanning electron microscopy (SEM) images, JEOL JSM-6700 scanning electron microscope was applied. The transmission electron microscopy (TEM) images were obtained on JEM-2100 transmission electron microscopy. The Electrochemistry experiments were conducted on a CHI660C Electrochemical Workstation (Shanghai Chenhua Electrochemical Instrument).

S1.2 Experimental Procedures

Synthesis of hexa(4-formyl-phenoxy)cyclotriphosphazene (CTP-6-CHO)

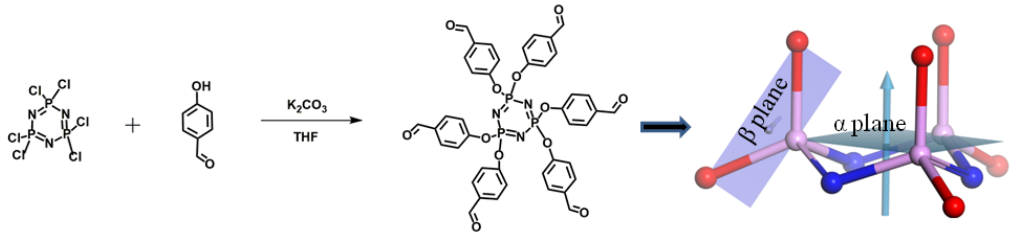

14.92 g *p*-hydroxybenzaldehyde was dissolved in 300 mL tetrahydrofuran, 33.4 g potassium carbonate was slowly added to the system, and stirred in an ice bath for 30 min. Then, 50 mL hexachlorocyclotriphosphazene (HCCP, 6.96 g) solution dissolved by tetrahydrofuran was slowly added, after 2 h of ice bath, stirring reaction was conducted at room temperature for 2 days. The reaction ends with filtration, decompression distillation of the filtrate, and the obtained solid was extracted with dichloromethane and washed with saturated salt water. The organic phase was dried with anhydrous magnesium sulfate. The white solid was obtained by decompression distillation, and the solid powder was recrystallized in ethyl acetate. The powder was dried at 50 ºC under vacuum overnight to obtain 14.98 g of white powder in ~87% yield [S1]. ^{1}H NMR (400 MHz, DMSO) δ 9.92 (s, 6H), 7.79 (d, 12H), 7.18 (d, 12H).

Synthesis of 1,3,5-tri(1,3-Diformyl biphenyl)benzene (TBPB-6-CHO)

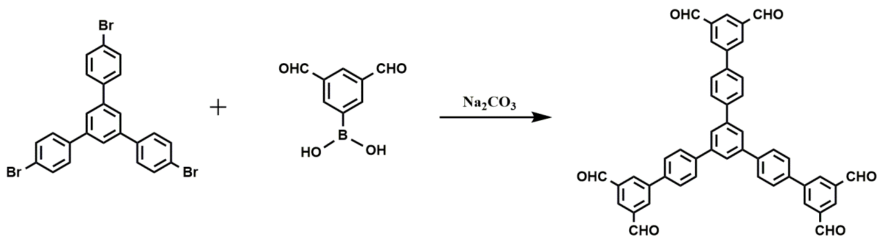

1,3,5-Tris(4-bromophenyl)benzene (1g, 1.84 mmol), (1,3-Diformyl biphenyl) boronic acid (994 mg, 6.62mmol) and Na_2CO_3 (320 mg, 15.9 mmol) were dissolved in a mixture of degassed toluene (20 mL), water (3.3 mL) and ethanol (6.6 mL) taken in a 100 mL Schlenck tube. Then the catalyst, $Pd(PPh_3)_4$ (138 mg, 0.119 mmol) was added under Argon flow and the mixture was degassed three times again. The resulting solution was then heated at 90 °C for 72 h. The combined layer was evaporated under reduced pressure. The crude product was purified directly without any workup by chromatography on silica gel using n-hexane/ EtOAc/acetone (7.5:1.5:1) as the eluent to give the title 1,3,5-tri(1,3-Diformyl biphenyl)benzene as an off-white solid (900 mg, 45%). ^{1}H NMR (400 MHz, $CDCl_3$): δ 10.22 (s, 6H); 8.43 (d, 6H); 8.39 (d, 3H); 7.94 (d, 6H); 7.90 (d, 3H); 7.84 (d, 6H).

2,4,6-Tris(4-aminophenyl)triazine (TAPT)

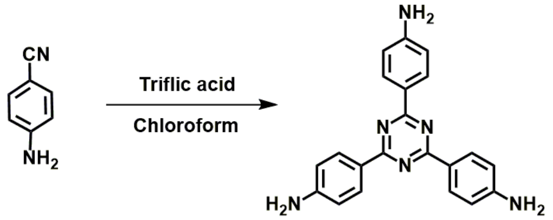

A suspension of 4-aminobenzonitrile (1.5 g, 12.70 mmol) in dry $CHCl_3$ (20 mL) in a 100 mL two-neck bottle was cooled in an ice bath under a flow of N_2 and then trifluoromethanesulfonic acid (4 mL, 0.045 mmol) was added. The mixture was stirred for 30 min in the ice bath and then warmed to room temperature. After stirring for an additional 24 h at room temperature, ice water was added. The aqueous suspension was neutralized by adding ammonium hydroxide. The precipitate was collected via vacuum filtration and dried under vacuum overnight to yield a white solid of TAPT [S2]. ^{1}H NMR (400 MHz, DMSO): δ 5.89 (s, 6H), 8.36 (d, 6H), 6.70 (d, 6H).

2,4,6-Tris(4-aminophenyl)benzene (TAPB)

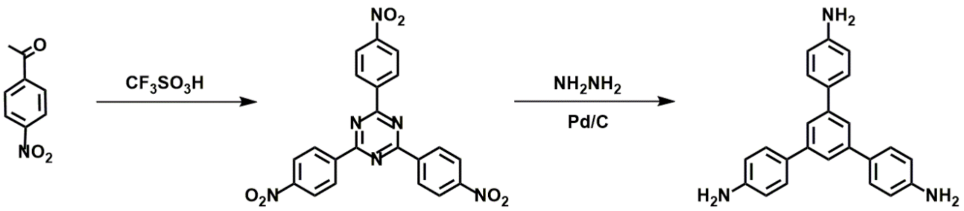

TAPB was synthesized according to the published procedures.2 4-Nitroacetophenone (25 g), toluene (100 mL), and CF_3SO_3H (1 mL) were added to a flask equipped with a water separator and a cooling condenser. The mixture was refluxed for 48 h, during this time the formed water was eliminated as a toluene azeotrope. After cooling down to room temperature, the mixture was filtered to yield a black solid product. It was washed with DMF under refluxing and filtered. This procedure was carried out twice

more, and a pale-yellow solid of 1,3,5-tris(4-nitrophenyl)benzene was obtained after drying. This product is insoluble in any common solvent and was used without further purification. A suspension of 1,3,5-tris(4-nitrophenyl)benzene (10 g, 22.7 mmol) and Pd/C (10 wt%, 2.0 g) in ethanol (200 mL) was heated to reflux. Hydrazine hydrate (30 mL) was added dropwise, and the mixture was refluxed overnight. The hot solution was filtered through celite and left undisturbed to fully crystallize the product. The solid was filtered and washed with cold ethanol to afford TAPB with 80% yield (6.36 g) [S3]. ^{1}H NMR (400 MHz, DMSO): δ 7.59 (s, 3H), 7.51 (d, 6H), 6.77 (d, 6H), 4.76 (s, 6H).

Synthesis of JUC-610, JUC-611 and JUC-612

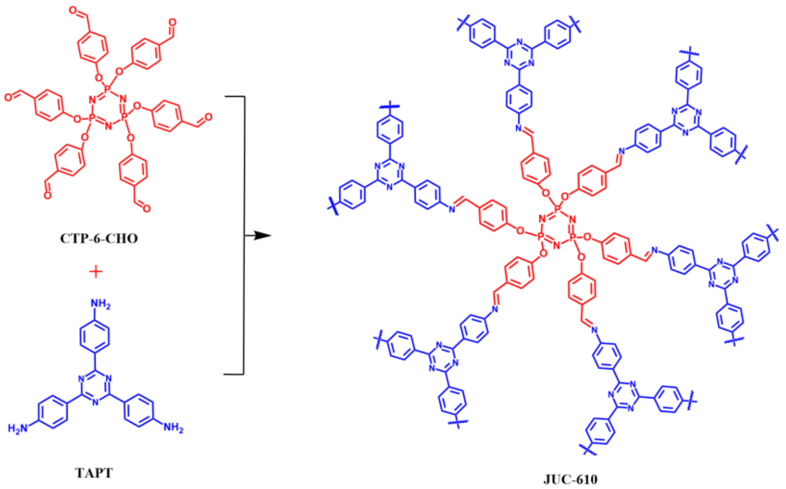

CTP-6-CHO (0.03 mmol, 28.07 mg) and TAPT (0.06 mmol, 20.12 mg) were weighted into a Pyrex tube (volume: ca. 20.0 ml with a body length of 18.0 cm and neck length of 9.0 cm). Then, the mixture of *o*-DCB (0.7 mL), n-BuOH (0.3 mL) and 0.1 ml of aqueous acetic acid (6.0 mol/L) was added. The Pyrex tube was flash frozen in a liquid nitrogen bath and evacuated to an internal pressure of ca. 19.0 mbar and flame-sealed, reducing the total length by ca. 10.0 cm. Upon warming to room temperature, the tube was placed in an oven at 120 °C for three days. As a result, a yellow powder was isolated by centrifugation and washed with acetone (3 × 5.0 ml) and the yield is about 75 %. Anal. Cald: C: 68.43; H: 3.67; N: 14.26; O: 7.33; P: 6.31. Found: C: 69.12; H: 3.81; N: 13.97; O: 7.21; P: 5.89. Solid-state ^{13}C NMR (500 MHz) δ (ppm): 165.05, 162.01, 147.46, 135.41, 125.51, 120.02, 115.98, 25.93. FT-IR: (KBr), ν/cm^{-1}: 1768.59, 1699.51, 1622.41, 1597.25, 1578.26, 1499.72, 1434.23, 1410.32, 1363.85, 1296.73, 1267.93, 1149.72, 952.98.

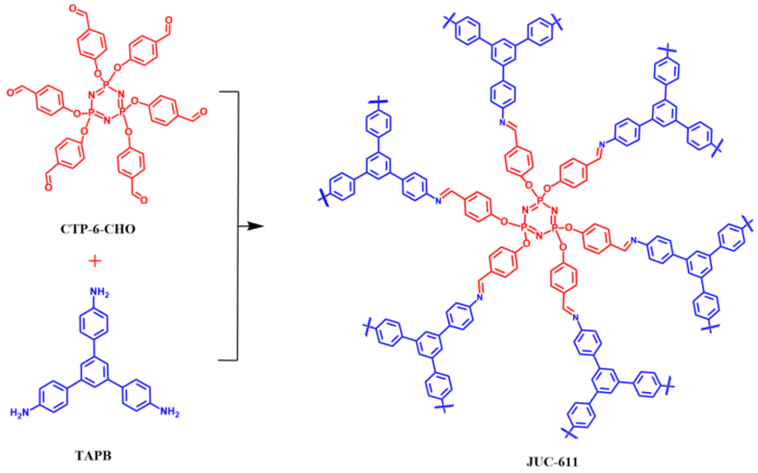

CTP-6-CHO (0.03 mmol, 28.07 mg) and TAPB (0.03 mmol, 20.10 mg) were weighted into a Pyrex tube (volume: ca. 20.0 ml with a body length of 18.0 cm and neck length of 9.0 cm). Then, the mixture of *o*-DCB (0.9 mL), n-BuOH (0.1 mL) and aqueous acetic acid (6.0 mol/L, 0.1 mL) was added. The Pyrex tube was flash frozen in a liquid nitrogen bath and evacuated to an internal pressure of ca. 19.0 mbar and flame-sealed, reducing the total length by ca. 10.0 cm. Upon warming to room temperature, the tube was placed in an oven at 120 °C for five days. As a result, a white powder was isolated by centrifugation and washed with acetone (3 × 5.0 ml) and the yield is about 72 %. Anal. Cald: C: 72.48; H: 4.23; N: 8.46; O: 7.25; P: 6.24. Found: C: 73.11; H: 4.01; N: 8.13; O: 7.78; P: 6.97. Solid-state ^{13}C NMR (500 MHz) δ (ppm): 161.32, 147.46, 142.35, 135.41, 12.93, 116.36, 25.18. FT-IR: (KBr), ν/cm^{-1}: 1702.95, 1624.34, 1595.92, 1506.25, 1296.73, 1269.56, 1202.44, 1176.51, 1155.93, 1098.36, 1015.21, 951.36.

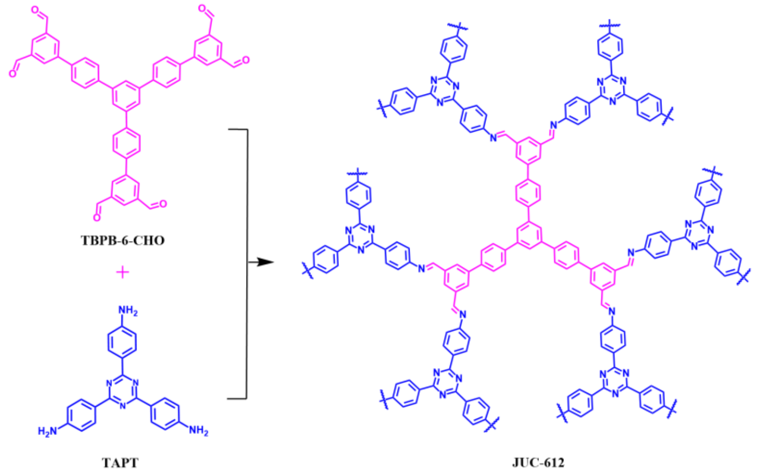

TBPB-6-CHO (0.03 mmol, 21.1 mg) and TAPT (0.03 mmol, 10.6 mg) were weighted into a Pyrex tube (volume: ca. 20.0 ml with a body length of 18.0 cm and neck length of 9.0 cm). Then, the mixture of Mesitylene (0.5 mL), 1, 4-Dioxane (0.5 mL) and 0.1 ml of aqueous acetic acid (6.0 mol/L) was added. The Pyrex tube was flash frozen in a liquid nitrogen bath and evacuated to an internal pressure of ca. 19.0 mbar and flame-sealed, reducing the total length by ca. 10.0 cm. Upon warming to room

temperature, the tube was placed in an oven at 120 °C for three days. As a result, a yellow powder was isolated by centrifugation and washed with acetone (3 × 5.0 ml) and the yield is about 79 %. Anal. Cald: C: 82.95; H: 4.15; N: 12.90. Found: C: 82.12; H: 4.52; N: 13.36. Solid-state ^{13}C NMR (500 MHz) δ (ppm): 185.93, 164.35, 146.01, 135.01, 131.38, 121.85, 25.55. FT-IR: (KBr), ν/cm^{-1}: 3034.48, 2806.27, 2725.08, 2308.15, 1695.92, 1581.18, 1508.67, 1404.81, 1368.97, 1140.75, 813.79.

Synthesis of JUC-610-CONs

In a typical experiment, 10.0 mg of bulk JUC-610 material was dispersed in 150 mL of ethanol. The mixture was sonicated in an ultrasonic bath (Brandson, CPX2800H-E, 110 W, 40 kHz) for 3 h. After sedimentation for 24 h, the upper colloidal suspension of exfoliated JUC-610-CONs was collected and concentrated by centrifugation at 7000 rmp for 5 min. After removal of supernatant, the residual JUC-610-CONs were redispersed into 20 mL of ethanol prior to characterization.

S1.3 Electrochemical Experiment

Oxygen Reduction Reaction measurements

The ORR catalytic activities of prepared four COFs catalysts were measured in 0.1 M KOH solution. An ink of the catalyst was prepared by mixing 3 mg of catalyst powder and 3 mg acetylene black with 250 μl of ethanol, 250 μl of H2O, and 25 μl 5 wt% Nafion solution, and further placed in an ultrasonic bath. 6 μL of the solution was loaded onto the electrode. The RDE measurements were performed in a three-electrode, one-compartment cell at room temperature, equipped with a graphite rod counter electrode and a Ag/AgCl reference electrode. Linear sweep voltammetry (LSV) was performed in N_2-saturated and O2-saturated 0.1 M KOH at a scan rate of 10 mV s^{-1} under various electrode rotation rates (400, 625, 900, 1025, 1600, 2025 and 2500 rpm, respectively). Long-term stability test was conducted by measuring the current changes of the catalyst at a fixed potential of 0.7 V (vs RHE) at a rotation speed of 1600 rpm in O_2-saturated electrolyte using on a CHI 760 E electrochemical workstation. Methanol tolerance tests were performed by chronoamperometric measurement at 0.7 V (vs RHE) at a rotating speed of 1600 rpm (methanol was dropped into the electrolytes at 200 s).

The turnover frequency (TOF) value is calculated according to the following equation:

$$TOF = (J \times A) / (4 \times F \times n)$$

where J is the current density at a given potential (0.7 V), A is the surface area of the electrode (0.0707 cm^2), the number of 4 represents 4 electrons/mol of O_2, F is the Faraday constant (96485.3 C/mol), and n stands for the number of moles of N, P atoms in samples.

For the zinc-air battery, the air electrodes were prepared by uniformly coating the as-prepared catalyst ink onto carbon paper and then drying it at 100 °C for 2 h. The mass loading was 1.0 mg cm^{-2}. A zinc plate was used as the anode. Both electrodes were assembled into a home-built electrochemical battery with the electrolyte being 6.0 M KOH.

S2 Characterization

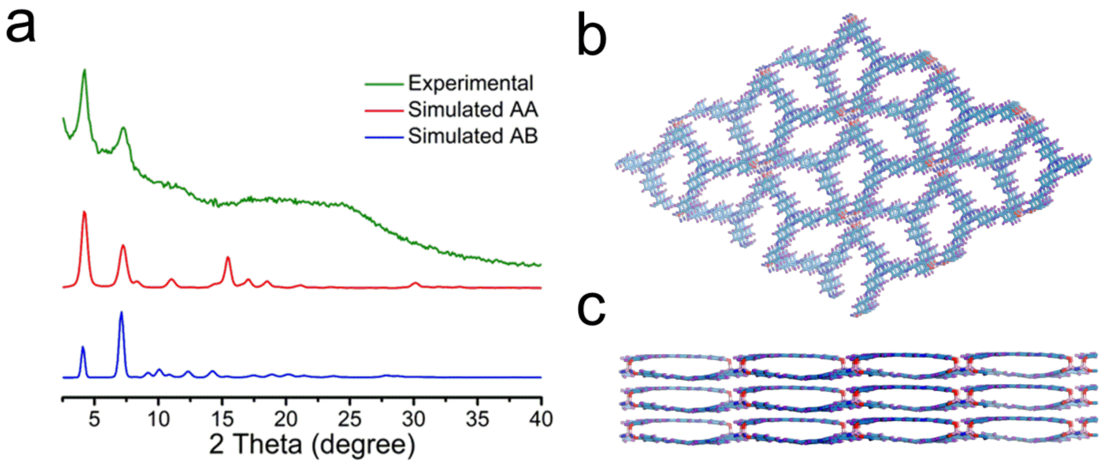

Fig. S1 (**a**) Comparison of PXRD patterns of JUC-610 (green), AA-stacking (red) and AB-stacking (blue)**;** (**b**) and (**c**) Top and side view of AA-stacking

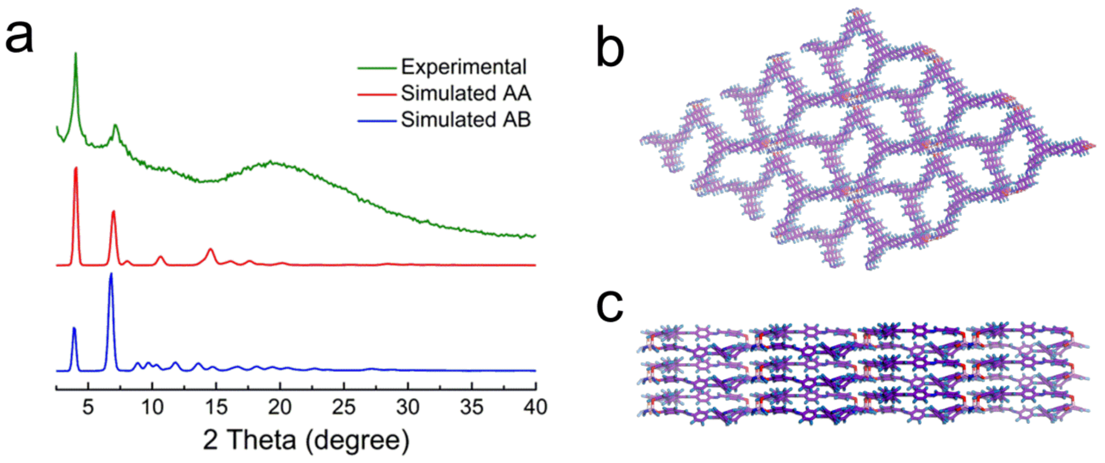

Fig. S2 (**a**) Comparison of PXRD patterns of JUC-611 (green), AA-stacking (red) and AB-stacking (blue)**;** (**b**) and (**c**) Top and side view of AA-stacking

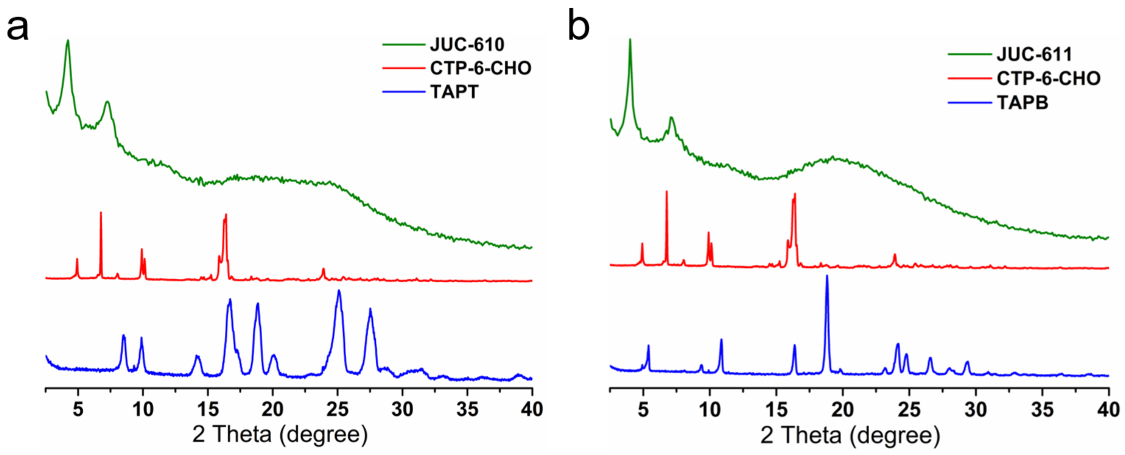

Fig. S3 (**a**) Comparison of PXRD patterns of JUC-610 (green) , CTP-6-CHO (red) and TAPT (blue) and (**b**) JUC-611 (green) , CTP-6-CHO (red) and TAPB (blue)

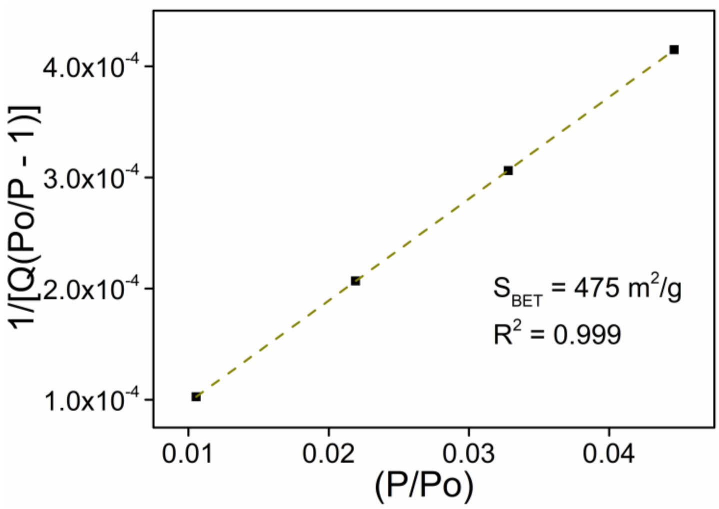

Fig. S4 BET plot of JUC-610 calculated from N_2 adsorption isotherm at 77 K

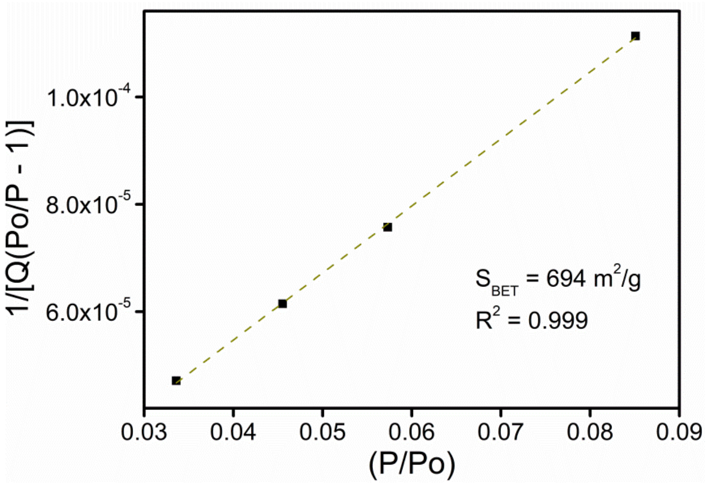

Fig. S5 BET plot of JUC-611 calculated from N_2 adsorption isotherm at 77 K

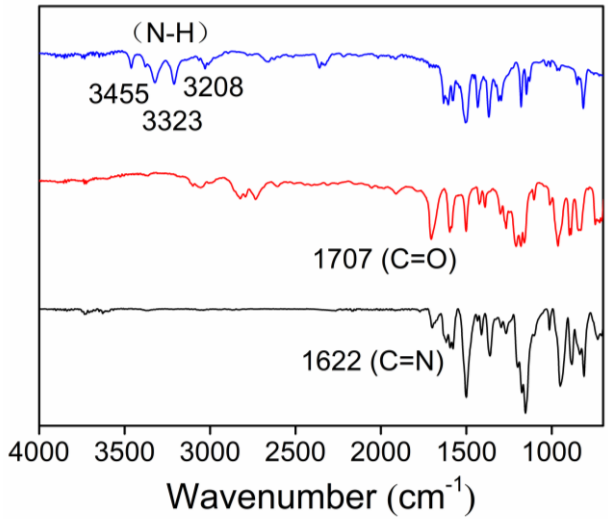

Fig. S6 FTIR spectra of JUC-610 (black), CTP-6-CHO (red) and TAPT (blue)

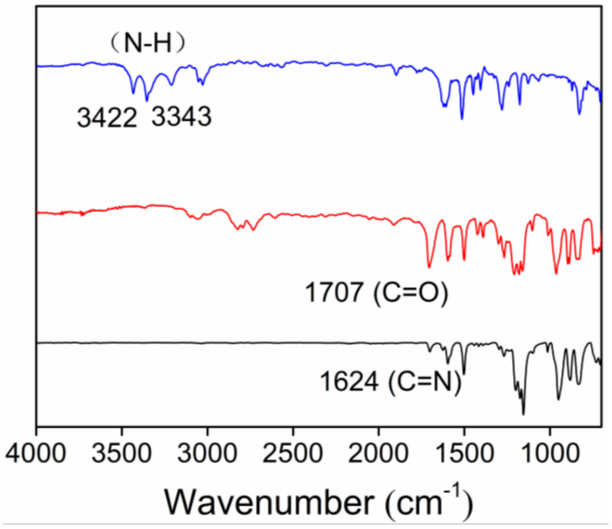

Fig. S7 FTIR spectra of JUC-611 (black), CTP-6-CHO (red) and TAPB (blue)

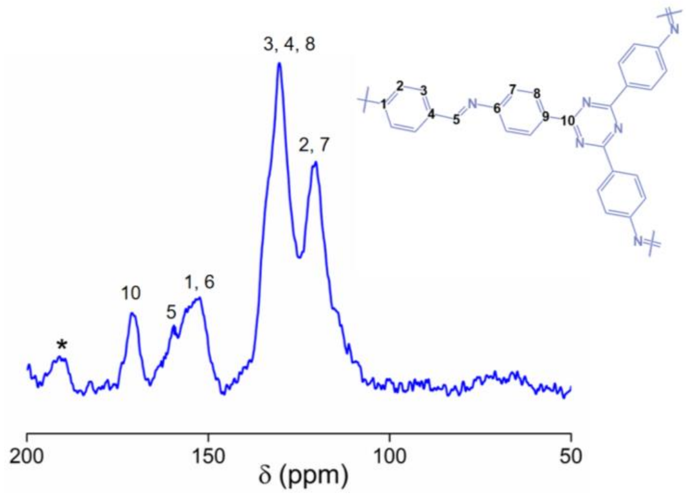

Fig. S8 ^{13}C CP/MS-NMR spectrum of JUC-610

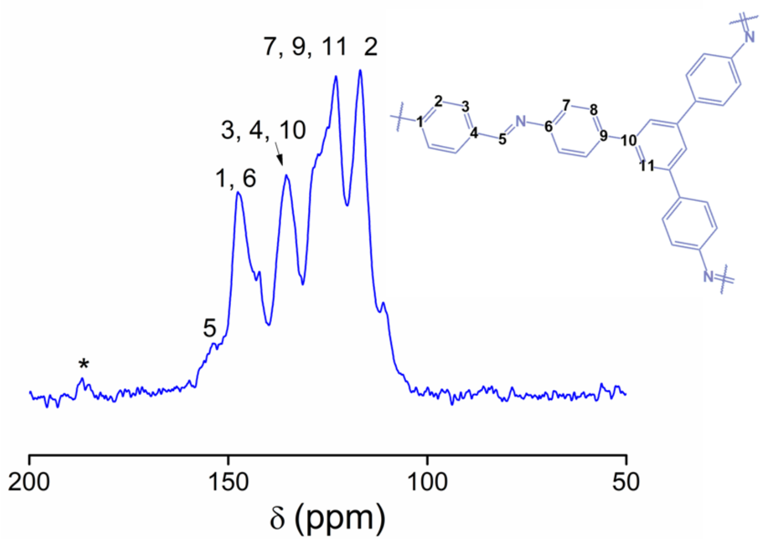

Fig. S9 ^{13}C CP/MS-NMR spectrum of JUC-611

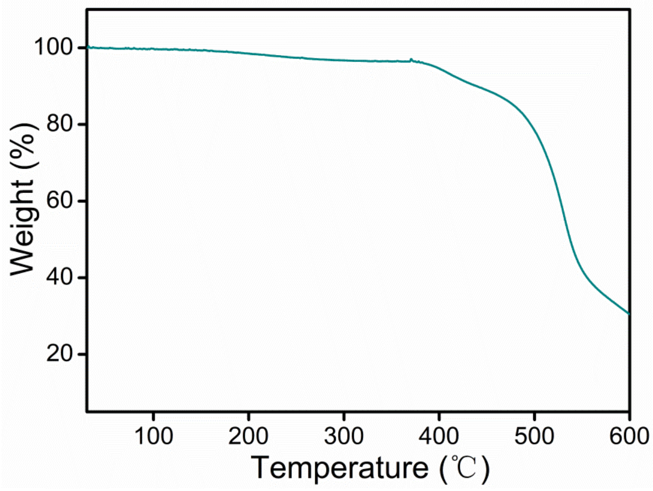

Fig. S10 TGA curve of JUC-610

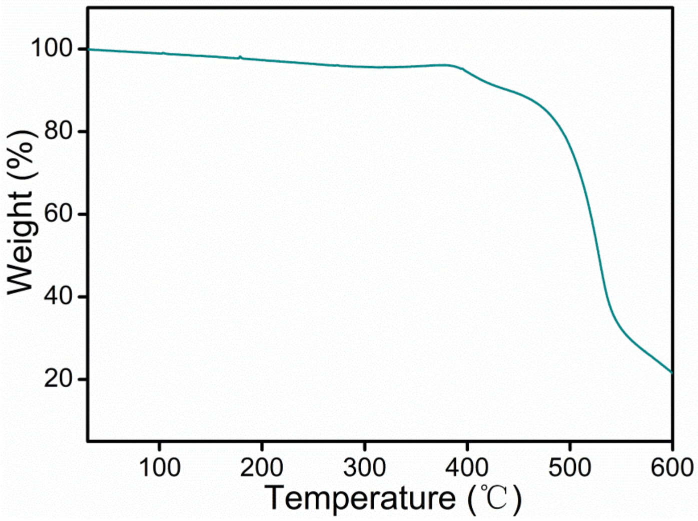

Fig. S11 TGA curve of JUC-611

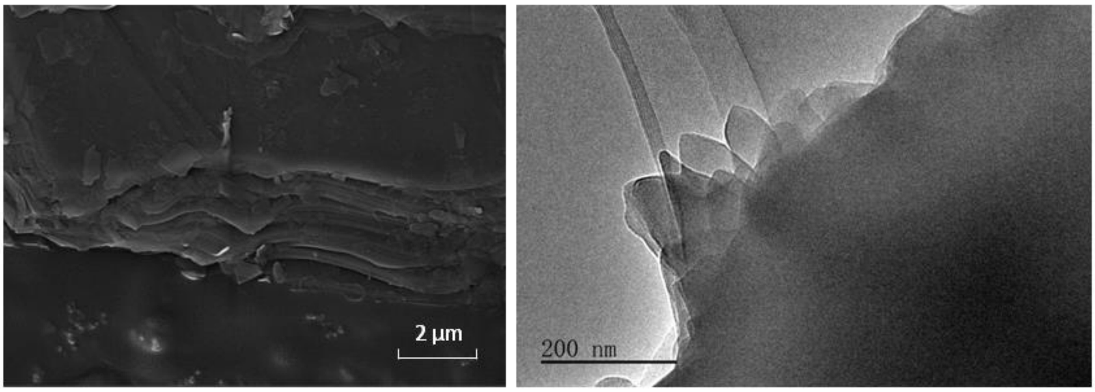

Fig. S12 SEM and TEM image of JUC-610

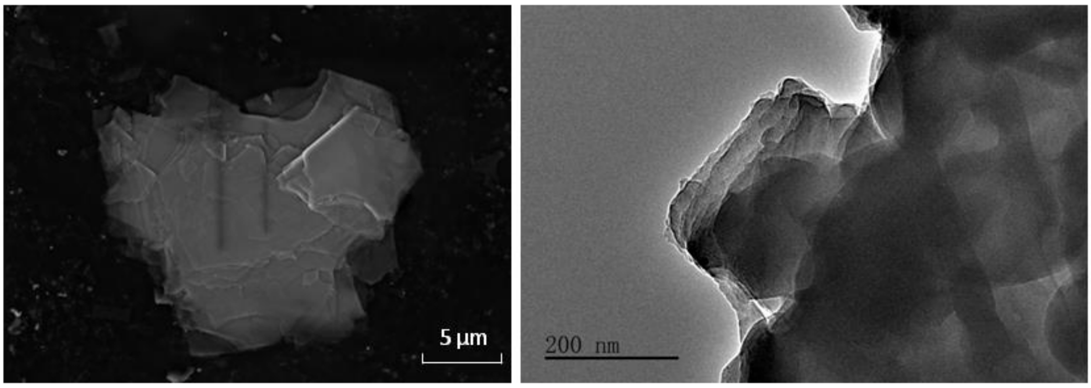

Fig. S13 SEM and TEM image of JUC-611

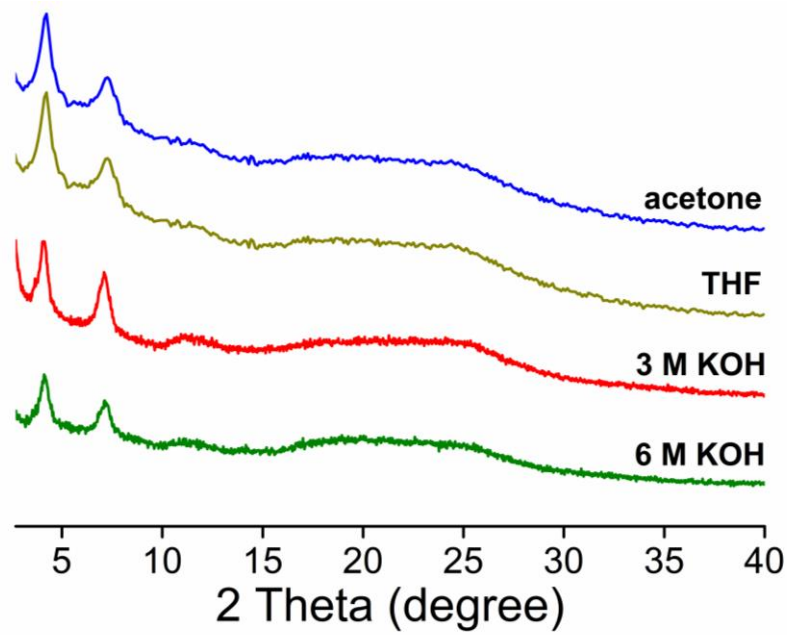

Fig. S14 PXRD patterns of JUC-610 after treatment under acetone, Tetrahydrofuran (THF), 3 M KOH and 6 M KOH for 24 h

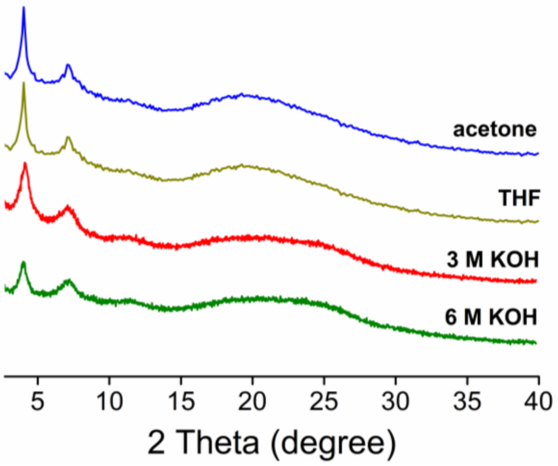

Fig. S15 PXRD patterns of JUC-611 after treatment under acetone, Tetrahydrofuran (THF), 3 M KOH and 6 M KOH for 24 h

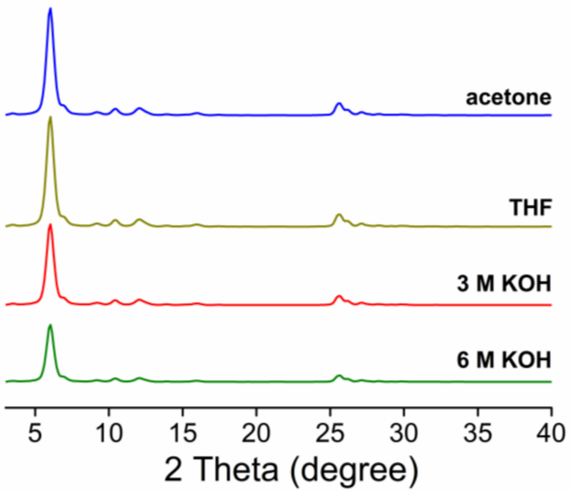

Fig. S16 PXRD patterns of JUC-612 after treatment under acetone, Tetrahydrofuran (THF), 3 M KOH and 6 M KOH for 24 h

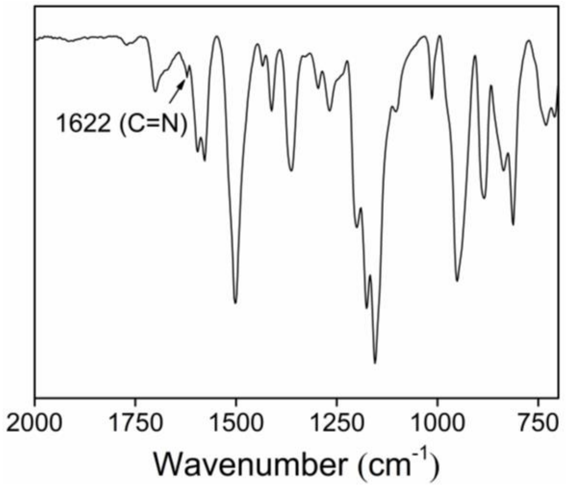

Fig. S17 FT-IR spectra of JUC-610 after treatment under 6 M KOH for 24 h

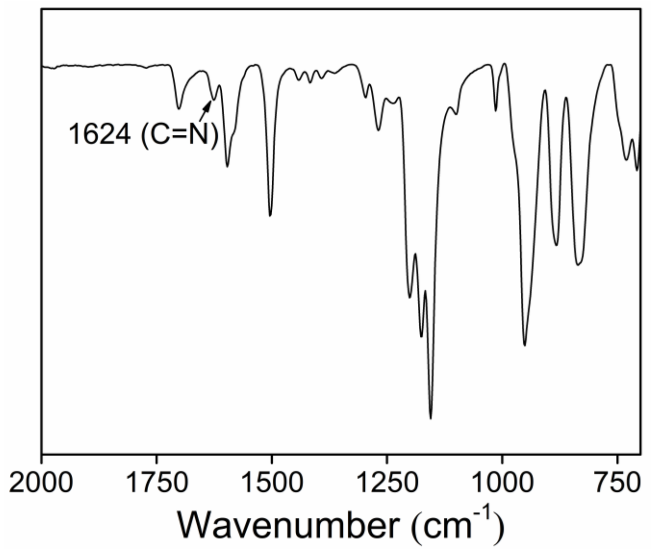

Fig. S18 FT-IR spectra of JUC-611 after treatment under 6 M KOH for 24 h

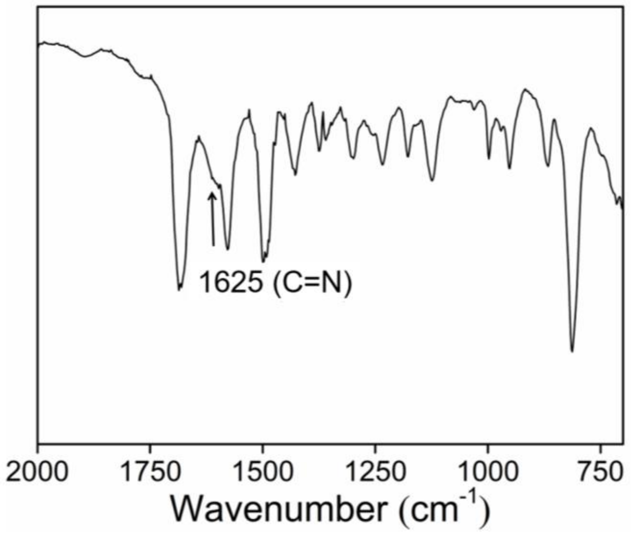

Fig. S19 FT-IR spectra of JUC-612 after treatment under 6 M KOH for 24 h

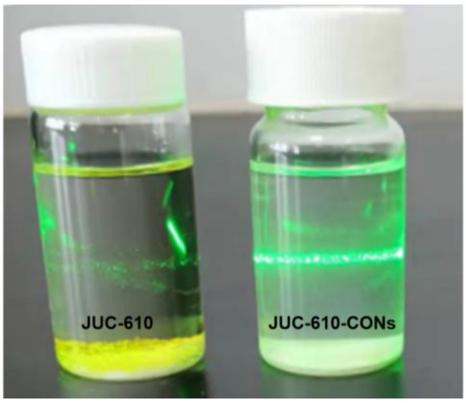

Fig. S20 Photograph of the Tyndall effect of JUC-610-CONs suspension and JUC-610 suspension obtained at the identified exfoliated conditions

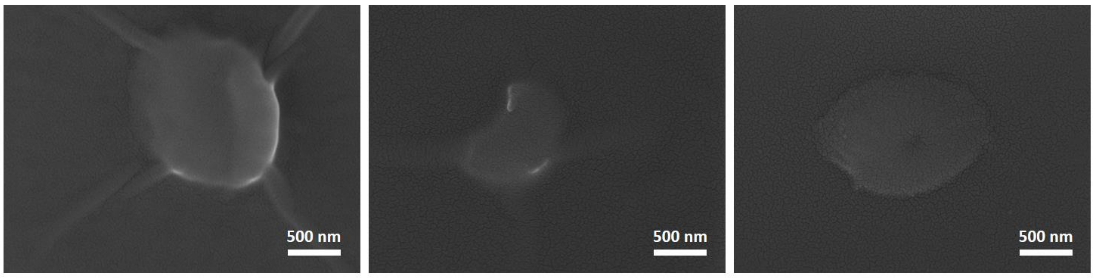

Fig. S21 SEM image of JUC-610-CON

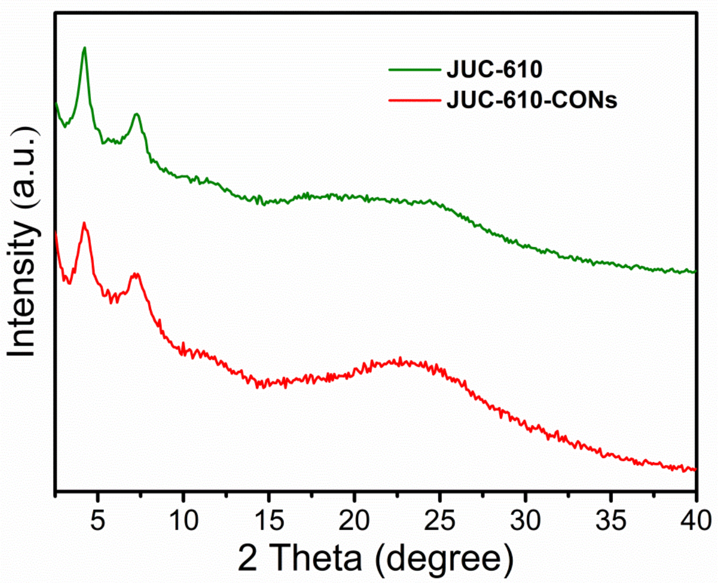

Fig. S22 Comparison of PXRD patterns of JUC-610 (green) and JUC-610-CONs (red)

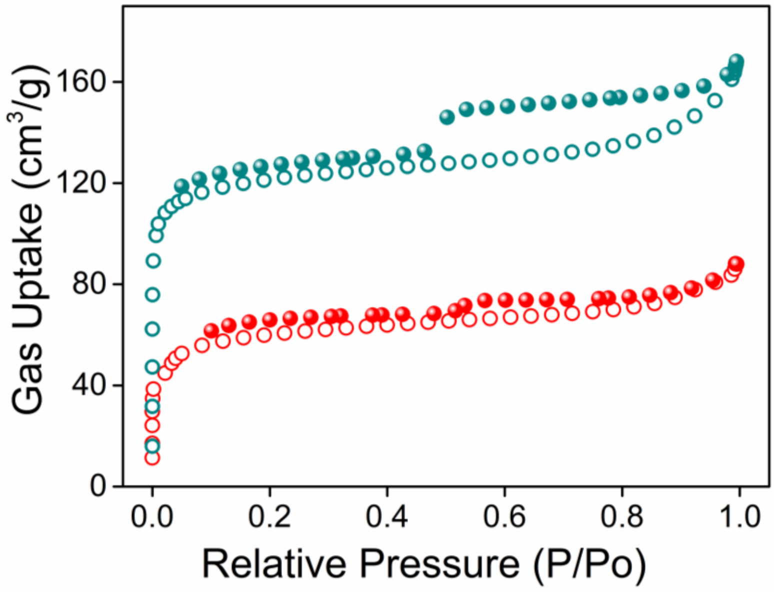

Fig. S23 N_2 adsorption−desorption isotherms for JUC-610 (green) and JUC-610-CONs (red)

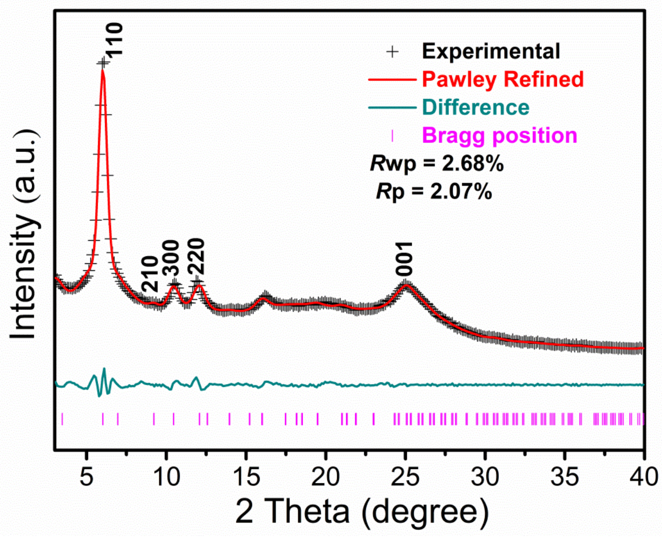

Fig. S24 PXRD patterns of JUC-612

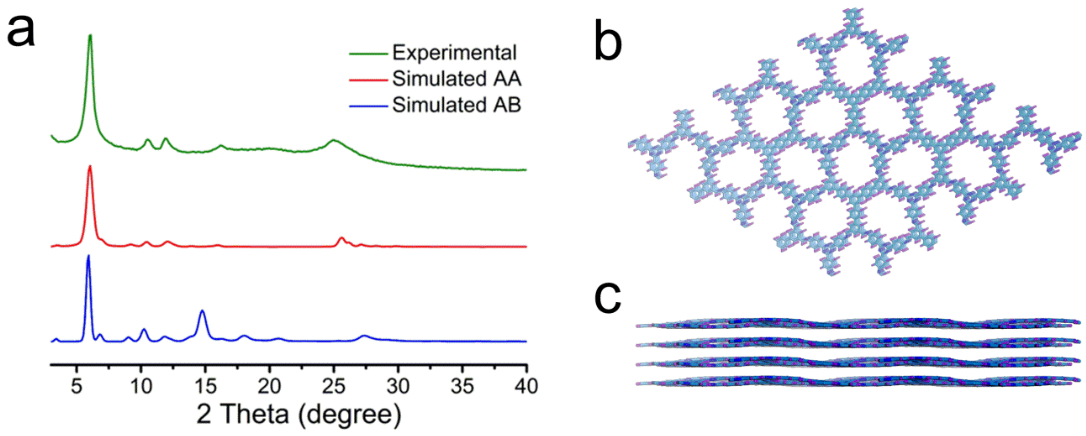

Fig. S25 (**a**) Comparison of PXRD patterns of JUC-612 (green), AA-stacking (red) and AB-stacking (blue) **;** (**b**) and (**c**) Top and side view of AA-stacking

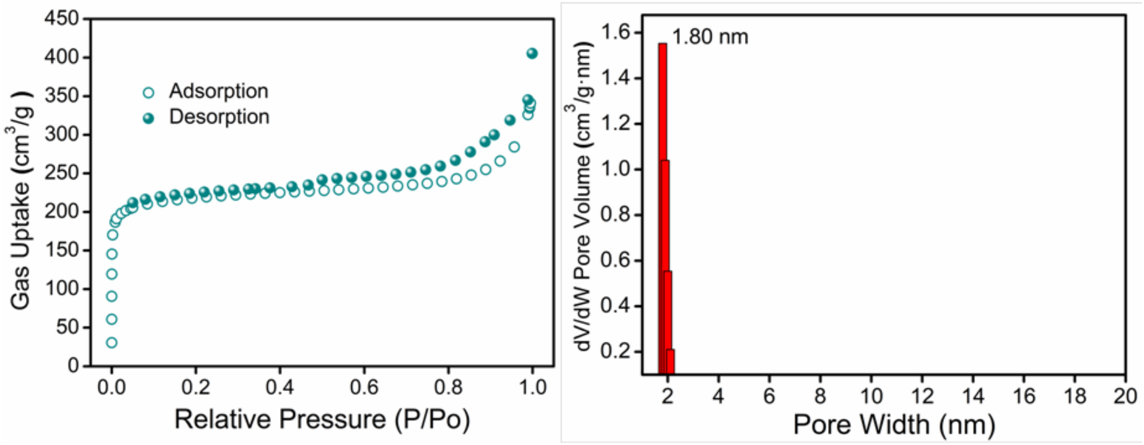

Fig. S26 N_2 adsorption−desorption isotherms and Pore-size distribution of calculated by fitting on the NLDFT model to the adsorption data for JUC-612

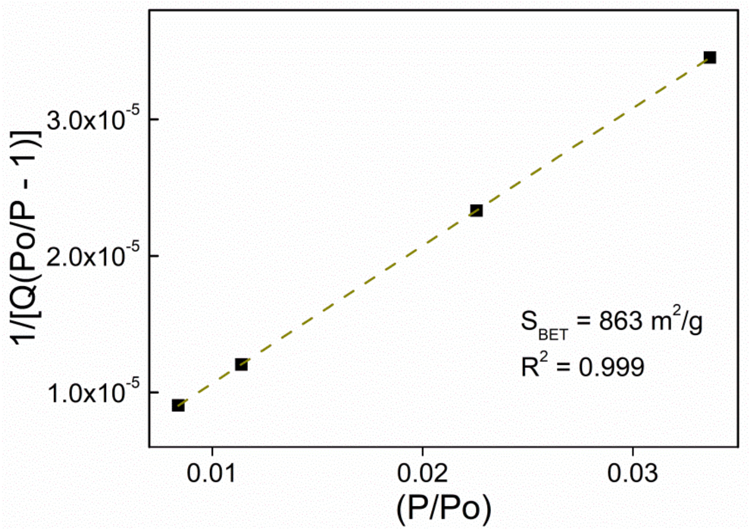

Fig. S27 BET plot of JUC-612 calculated from N_2 adsorption isotherm at 77 K

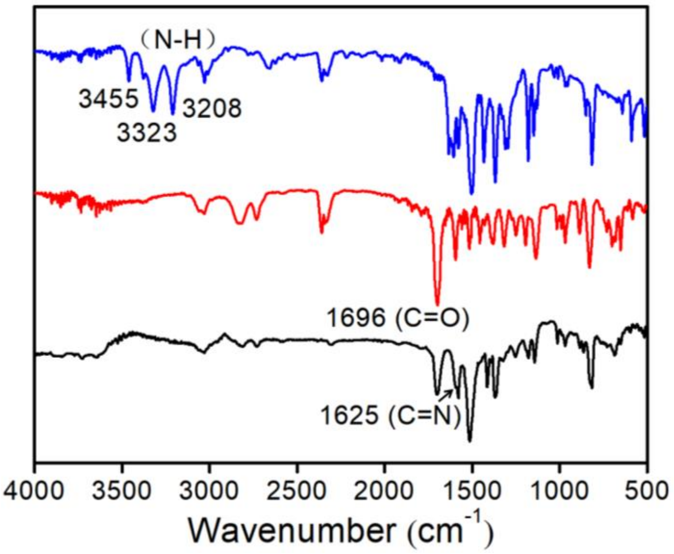

Fig. S28 FTIR spectra of JUC-612 (black), TDBPB (red) and TAPT (blue)

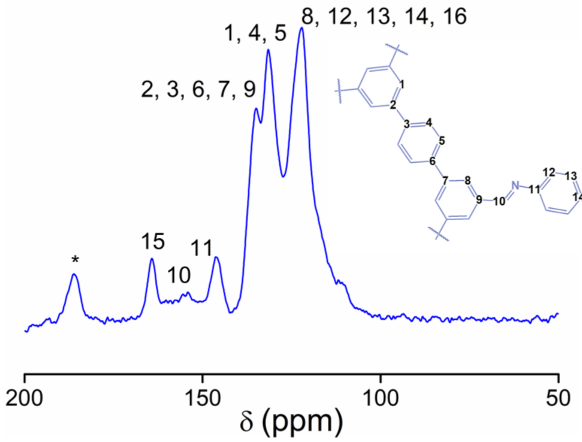

Fig. S29 ^{13}C CP/MS-NMR spectrum of JUC-612

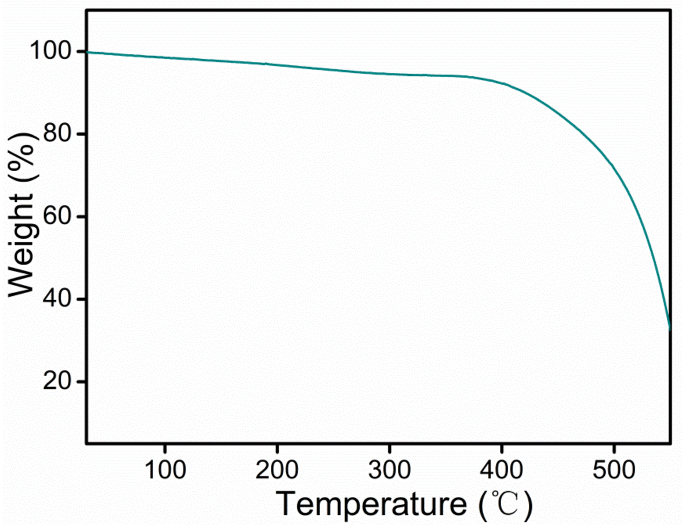

Fig. S30 TGA curve of JUC-612

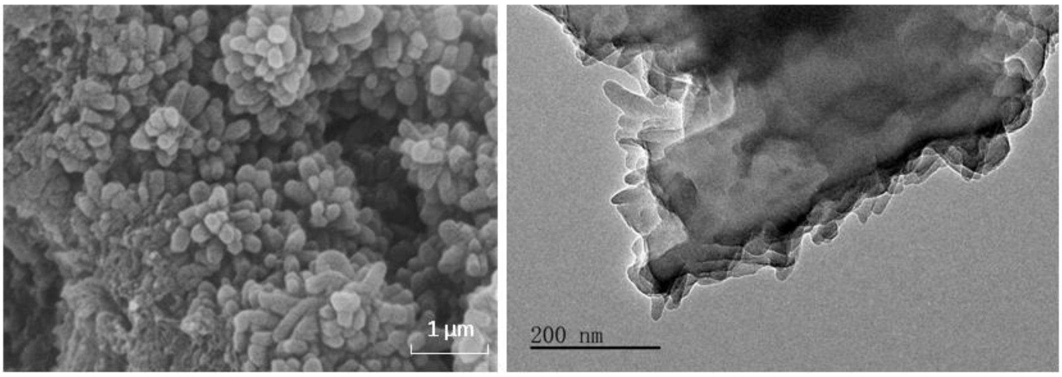

Fig. S31 SEM and TEM image of JUC-612

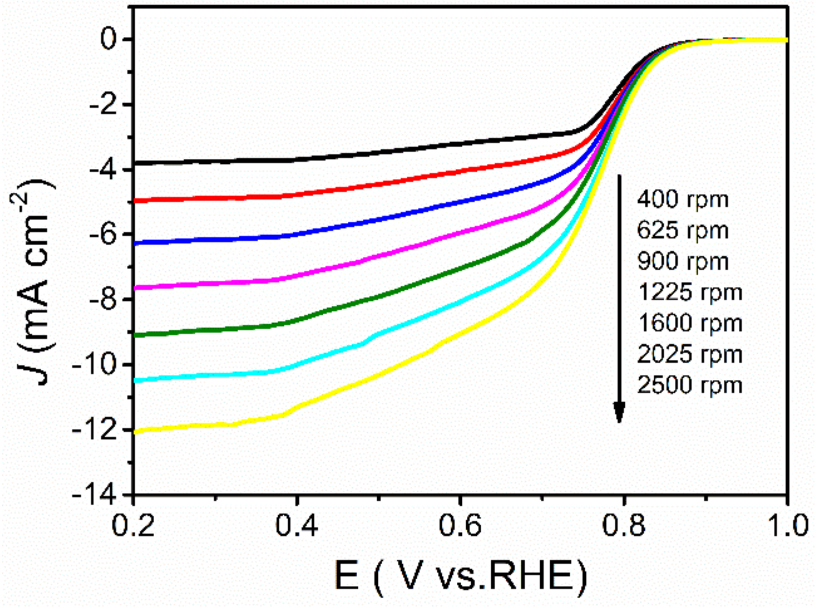

Fig. S32 LSV curves of JUC-610-CONs at various rotation speeds

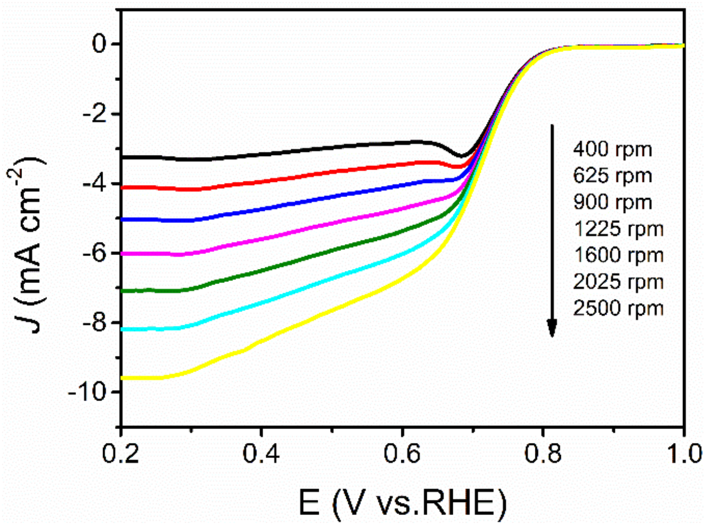

Fig. S33 LSV curves of JUC-610 at various rotation speeds

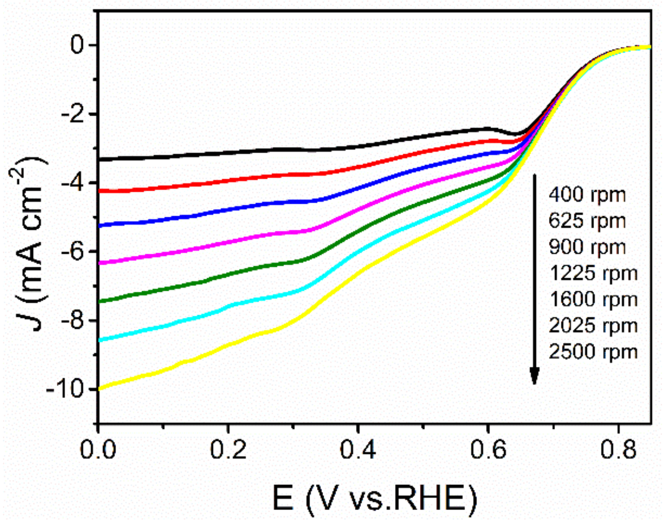

Fig. S34 LSV curves of JUC-611 at various rotation speeds

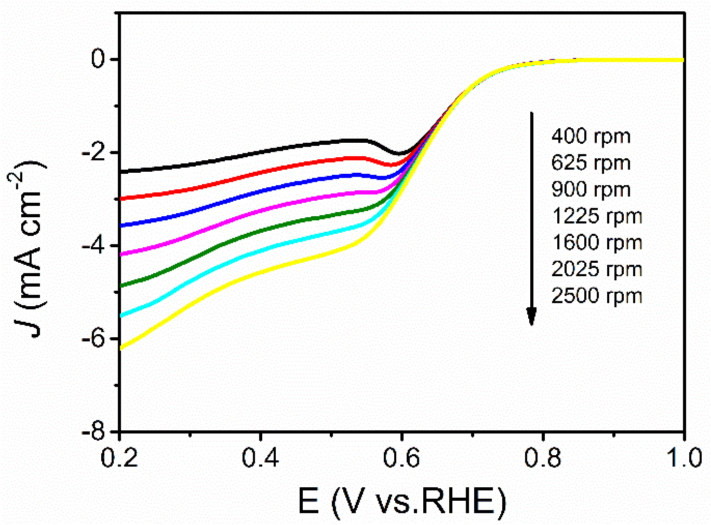

Fig. S35 LSV curves of JUC-612 at various rotation speeds

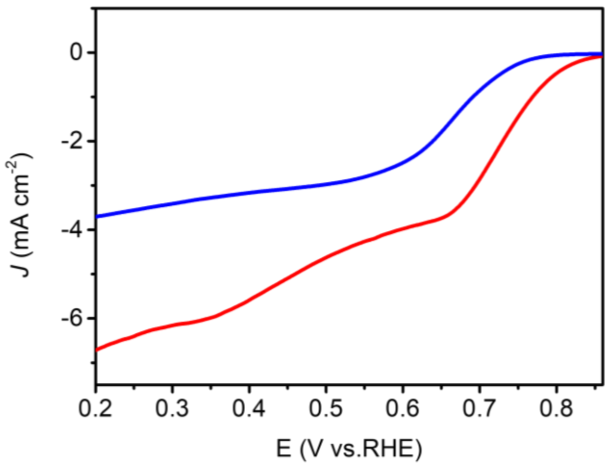

Fig. S36 LSV curves of JUC-610 (red) and physical mixing of two monomers (blue) at 1600 rpm in O2-saturated 0.1 M KOH electrolyte

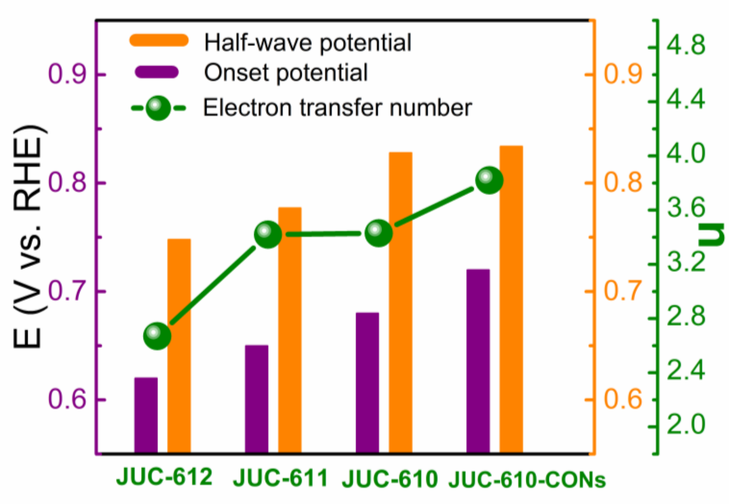

Fig. S37 Comparison of onset potential, half-wave potential and electron transfer number of JUC-610-CONs, JUC-610, JUC-611 and JUC-612

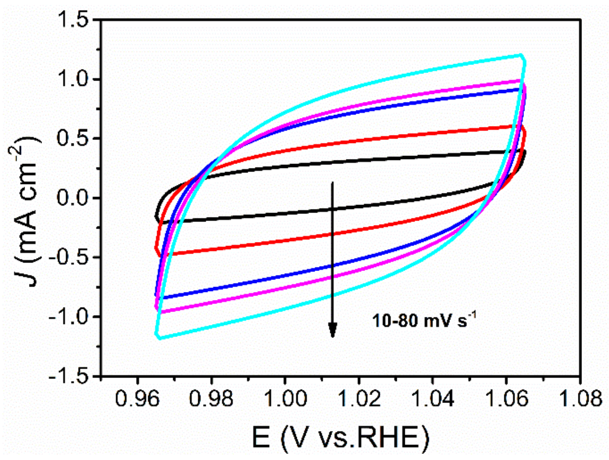

Fig. S38 CVs of JUC-610-CONs in 0.1 M KOH solution at different scan rates (10, 20, 40, 60, and 80 mV s^{-1})

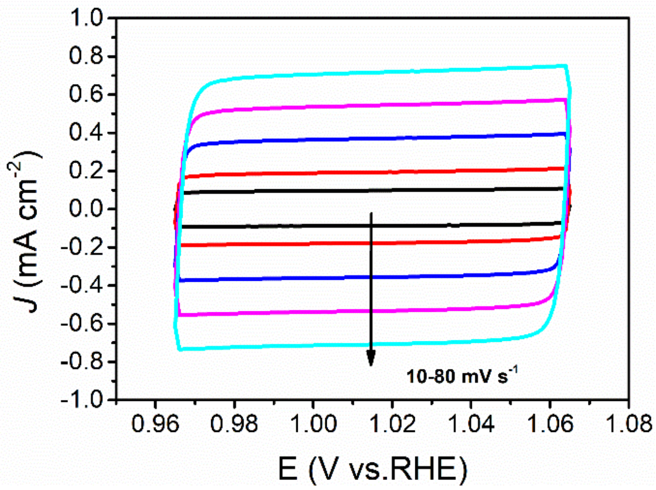

Fig. S39 CVs of JUC-610 in 0.1 M KOH solution at different scan rates (10, 20, 40, 60, and 80 mV s^{-1})

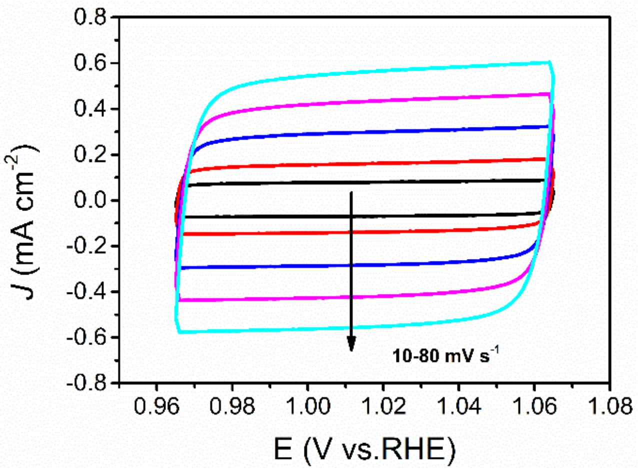

Fig. S40 CVs of JUC-611 in 0.1 M KOH solution at different scan rates (10, 20, 40, 60, and 80 mV s^{-1})

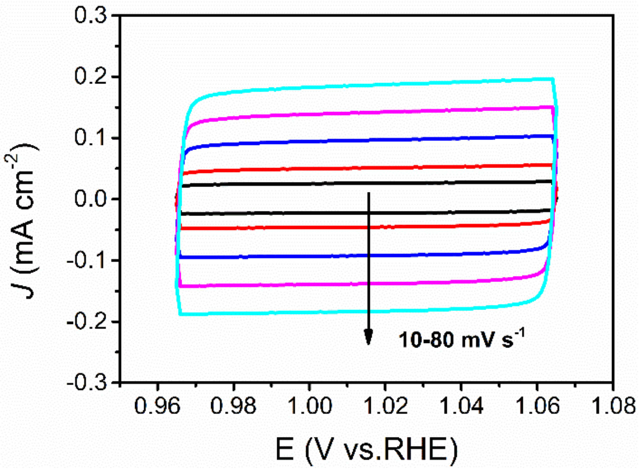

Fig. S41 CVs of JUC-612 in 0.1 M KOH solution at different scan rates (10, 20, 40, 60, and 80 mV s^{-1})

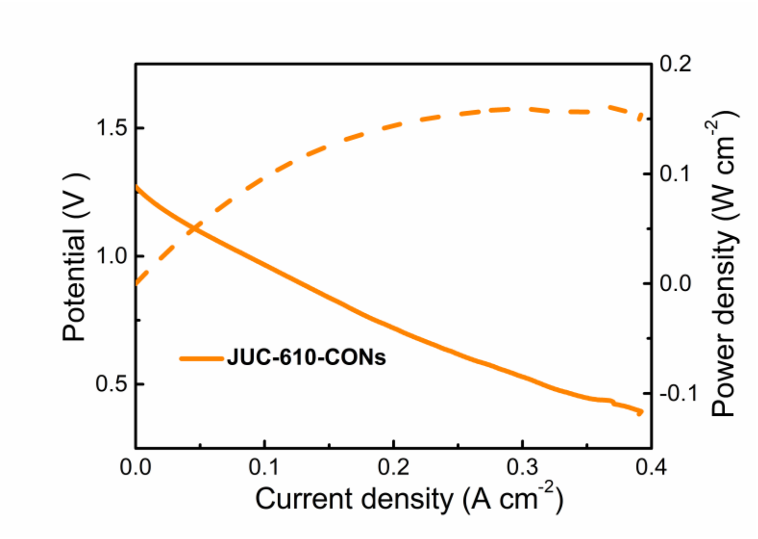

Fig. S42 The discharge polarization curves and corresponding power density plots of JUC-610-CON-based ZAB

Table S1 Unit cell parameters and fractional atomic coordinates for JUC-610 calculated based on the eclipsed ***hcb*** net

Space group		*P*3	
Calculated unit cell		$a = b = 24.6620$ Å, $c = 5.9759$ Å, $\alpha = \beta = 90°$, $\gamma = 120°$	
Measured unit cell		$a = b = 24.6869$ Å, $c = 5.9497$ Å, $\alpha = \beta = 90°$, $\gamma = 120°$	
Pawley refinement		*R*p = 1.29%, *R*wp = 1.78%	
atoms	x	y	z
C1	0.32029	0.71029	-0.66283
N2	0.37673	0.72308	-0.66228
C3	0.46586	0.6499	-0.69764
C4	0.52371	0.66367	-0.6692
C5	0.56707	0.72147	-0.63505
C6	0.55217	0.76552	-0.6335
C7	0.49469	0.75184	-0.66259
C8	0.45106	0.69396	-0.6938
C9	0.66502	0.78664	-0.59922
N10	0.62587	0.73493	-0.65578
C11	0.7473	0.7654	-0.60165
C12	0.8071	0.78562	-0.58303
C13	0.84573	0.84379	-0.53211
C14	0.82322	0.88097	-0.49563

C15	0.76395	0.86112	-0.51372
C16	0.72594	0.80362	-0.56865
O17	0.90583	0.86347	-0.52956
P18	0.95106	0.93017	-0.40411
C19	0.69501	0.30211	0.00701
N20	0.63565	0.27418	0.00687
C21	0.51302	0.30598	-0.00503
C22	0.45201	0.27713	-0.0124
C23	0.41929	0.21613	-0.01115
C24	0.44857	0.18415	-0.00197
C25	0.50965	0.21305	0.0052
C26	0.54247	0.27427	0.00334
C27	0.32258	0.13185	-0.02153
N28	0.35684	0.18806	-0.02419
C29	0.23217	0.13902	-0.06553
C30	0.17175	0.11225	-0.09303
C31	0.1373	0.05124	-0.09457
C32	0.16373	0.01732	-0.06551
C33	0.22422	0.04415	-0.04012
C34	0.25883	0.10503	-0.04085
O35	0.07825	0.0246	-0.14458
N36	0.04659	0.06627	-0.48191
H37	0.43045	0.6027	-0.7241
H38	0.53554	0.62775	-0.67387
H39	0.58733	0.81288	-0.60826
H40	0.483	0.78786	-0.66105
H41	0.64989	0.81957	-0.5732
H42	0.71616	0.71799	-0.64353
H43	0.82464	0.75464	-0.60938
H44	0.85373	0.92822	-0.45042
H45	0.74644	0.89194	-0.48356
H46	0.53925	0.3557	-0.00581
H47	0.42856	0.30336	-0.01948
H48	0.42262	0.13443	-0.00033
H49	0.53312	0.18685	0.01262
H50	0.34306	0.1031	-0.00367
H51	0.25995	0.18864	-0.06313

H52	0.15037	0.14008	-0.11427
H53	0.13597	-0.0323	-0.06247
H54	0.24565	0.01637	-0.01865

Table S2 Unit cell parameters and fractional atomic coordinates for JUC-610 calculated based on the staggered ***hcb*** net

Space group		*P*3	
Calculated unit cell		$a = b = 24.8855$ Å, $c = 9.6139$ Å, $\alpha = \beta = 90°$, $\gamma = 120°$	
atoms	x	y	z
C1	0.32411	0.71309	0.11069
N2	0.37955	0.72215	0.11091
C3	0.46034	0.6393	0.1146
C4	0.51802	0.65012	0.12557
C5	0.56435	0.70767	0.14272
C6	0.55246	0.75416	0.14754
C7	0.4954	0.74347	0.13556
C8	0.44898	0.68612	0.11871
C9	0.6649	0.77101	0.1834
N10	0.62363	0.71939	0.14666
C11	0.74915	0.75647	0.11768
C12	0.81003	0.78025	0.10555
C13	0.84864	0.837	0.15468
C14	0.82513	0.869	0.21759
C15	0.76507	0.84571	0.22958
C16	0.72699	0.78969	0.17969
O17	0.9094	0.8608	0.13558
P18	0.95445	0.92939	0.2184
C19	0.69311	0.29998	0.48239
N20	0.63352	0.27392	0.48242
C21	0.51432	0.31198	0.47956
C22	0.45298	0.28482	0.48094
C23	0.41844	0.22358	0.48736
C24	0.44608	0.18965	0.49021
C25	0.5074	0.21683	0.48791
C26	0.54208	0.27823	0.48328
C27	0.32022	0.14199	0.51309

N28	0.35567	0.19691	0.484
C29	0.23134	0.14625	0.43758
C30	0.17102	0.11803	0.41089
C31	0.13486	0.05853	0.44525
C32	0.15932	0.02847	0.51201
C33	0.21978	0.05668	0.5381
C34	0.2562	0.11535	0.499
O35	0.07618	0.02781	0.40235
N36	0.04373	0.06746	0.1616
C37	0.64875	0.3738	0.84484
N38	0.70688	0.39137	0.84407
C39	0.80686	0.32952	0.82082
C40	0.86381	0.34611	0.77701
C41	0.90153	0.40345	0.73117
C42	0.88248	0.44474	0.73473
C43	0.82582	0.42824	0.77887
C44	0.78728	0.37033	0.82029
C45	0.99408	0.47058	0.63049
N46	0.95775	0.41822	0.67533
C47	0.06587	0.44326	0.53488
C48	0.12211	0.46032	0.48445
C49	0.1641	0.51997	0.4716
C50	0.14796	0.56221	0.50301
C51	0.0919	0.5453	0.55207
C52	0.05113	0.48598	0.57099
O53	0.22166	0.53572	0.43349
P54	0.27673	0.60005	0.51642
C55	0.02806	0.96811	0.80893
N56	0.96832	0.94046	0.80884
C57	0.84577	0.9733	0.78623
C58	0.78433	0.94438	0.77991
C59	0.75099	0.88326	0.79423
C60	0.78014	0.85132	0.81517
C61	0.84158	0.88026	0.82105
C62	0.87502	0.9415	0.80629
C63	0.65348	0.79884	0.80245
N64	0.68812	0.85473	0.78234

C65	0.56225	0.80129	0.73484
C66	0.50149	0.7719	0.71109
C67	0.46708	0.71136	0.73737
C68	0.49403	0.68104	0.78976
C69	0.55479	0.71032	0.81199
C70	0.58933	0.77039	0.78414
O71	0.40767	0.68014	0.69969
N72	0.38765	0.73035	0.46077
H73	0.42033	0.58975	0.10161
H74	0.52769	0.60997	0.12024
H75	0.59202	0.80382	0.16217
H76	0.48577	0.78367	0.13968
H77	0.64912	0.80419	0.22226
H78	0.71608	0.70811	0.07552
H79	0.82924	0.75225	0.05331
H80	0.85759	0.91718	0.26148
H81	0.74602	0.87368	0.28266
H82	0.54371	0.36472	0.47516
H83	0.42958	0.31417	0.47657
H84	0.41699	0.13688	0.49464
H85	0.53082	0.18748	0.4899
H86	0.3408	0.11288	0.55222
H87	0.26241	0.19709	0.40829
H88	0.15022	0.14462	0.35873
H89	0.12818	-0.02161	0.54675
H90	0.24033	0.03065	0.59381
H91	0.77476	0.28017	0.85892
H92	0.88065	0.31111	0.77855
H93	0.91492	0.49453	0.69975
H94	0.80967	0.46377	0.78161
H95	0.97935	0.50708	0.63925
H96	0.03019	0.39194	0.54722
H97	0.13425	0.42358	0.45221
H98	0.18256	0.61366	0.48778
H99	0.07882	0.58201	0.578
H100	0.87401	1.02589	0.77443
H101	0.75977	0.97207	0.76205

H102	0.75225	0.79877	0.82817
H103	0.86619	0.8526	0.83891
H104	0.67526	0.77018	0.83704
H105	0.5916	0.85315	0.71318
H106	0.4788	0.79848	0.66823
H107	0.46485	0.62959	0.81579
H108	0.57753	0.68385	0.8552

Table S3 Unit cell parameters and fractional atomic coordinates for JUC-611 calculated based on the eclipsed ***hcb*** net

Space group		*P*3	
Calculated unit cell		$a = b = 25.6008$ Å, $c = 6.4398$ Å, $\alpha = \beta = 90°$, $\gamma = 120°$	
Measured unit cell		$a = b = 25.5967$ Å, $c = 6.4417$ Å, $\alpha = \beta = 90°$, $\gamma = 120°$	
Pawley refinement		*R*p = 1.54%, *R*wp = 2.09%	
atoms	x	y	z
C1	0.32861	0.71727	-0.69821
C2	0.38352	0.72163	-0.69851
C3	0.45748	0.6413	-0.55618
C4	0.51412	0.65142	-0.52862
C5	0.56065	0.69752	-0.6293
C6	0.5498	0.73061	-0.76934
C7	0.49339	0.7202	-0.79875
C8	0.44692	0.67677	-0.68744
C9	0.65506	0.76687	-0.51951
N10	0.6193	0.71401	-0.57789
C11	0.74335	0.75496	-0.53838
C12	0.80405	0.78078	-0.54305
C13	0.83983	0.84051	-0.50621
C14	0.81346	0.87308	-0.44695
C15	0.75315	0.84739	-0.44131
C16	0.71768	0.78888	-0.49406
O17	0.89996	0.865	-0.54168
P18	0.94846	0.93169	-0.42131
C19	0.69766	0.30267	-0.04026
C20	0.63632	0.27235	-0.03986

C21	0.50946	0.28897	-0.1664
C22	0.44852	0.25814	-0.17256
C23	0.41656	0.20705	-0.06016
C24	0.44682	0.18775	0.06315
C25	0.50787	0.21871	0.06989
C26	0.54	0.26964	-0.0453
C27	0.31792	0.12383	-0.0184
N28	0.35388	0.17731	-0.07221
C29	0.22965	0.13283	-0.09735
C30	0.16945	0.10727	-0.12595
C31	0.13279	0.04716	-0.09731
C32	0.15661	0.01343	-0.03075
C33	0.21697	0.03856	-0.00842
C34	0.25413	0.09846	-0.04117
O35	0.07428	0.02003	-0.15548
N36	0.04906	0.06487	-0.50412
H37	0.42643	0.76825	-0.69902
H38	0.41792	0.60273	-0.46909
H39	0.52261	0.62097	-0.42015
H40	0.58893	0.76759	-0.86289
H41	0.48443	0.74799	-0.91791
H42	0.63549	0.79779	-0.48633
H43	0.71356	0.70434	-0.57193
H44	0.82588	0.752	-0.57859
H45	0.84291	0.92288	-0.4014
H46	0.73135	0.87519	-0.39136
H47	0.61011	0.22053	-0.03917
H48	0.53599	0.33186	-0.26371
H49	0.42324	0.27512	-0.27369
H50	0.42046	0.14509	0.16168
H51	0.5331	0.20216	0.17325
H52	0.33688	0.09528	0.04976
H53	0.26043	0.18385	-0.12049
H54	0.14914	0.13651	-0.17475
H55	0.12553	-0.03673	0.00699
H56	0.23725	0.00916	0.03868
H57	0.07631	0.10418	-0.60795

Table S4 Unit cell parameters and fractional atomic coordinates for JUC-611 calculated based on the staggered ***hcb*** net

Space group		*P3*	
Calculated unit cell		$a = b = 26.0966$ Å, $c = 9.9674$ Å, $\alpha = \beta = 90°$, $\gamma = 120°$	
atoms	x	y	z
C1	0.32391	0.7156	0.06254
C2	0.38145	0.72412	0.05999
C3	0.4677	0.64266	0.04365
C4	0.52432	0.6514	0.06733
C5	0.5675	0.70348	0.1275
C6	0.55353	0.74704	0.16362
C7	0.49705	0.73813	0.14235
C8	0.45299	0.68602	0.08046
C9	0.66187	0.74609	0.23593
N10	0.62673	0.71419	0.14017
C11	0.75086	0.74516	0.14266
C12	0.81214	0.77437	0.12279
C13	0.84905	0.82972	0.18156
C14	0.82313	0.85417	0.26583
C15	0.76261	0.82419	0.28928
C16	0.72589	0.77045	0.22533
O17	0.90992	0.8583	0.1502
P18	0.95556	0.92914	0.22138
C19	0.695	0.2993	0.50861
C20	0.63295	0.27157	0.5086
C21	0.50916	0.29799	0.43287
C22	0.44747	0.27026	0.43435
C23	0.41376	0.21923	0.51099
C24	0.44307	0.19624	0.58753
C25	0.50482	0.22404	0.58611
C26	0.53861	0.27515	0.50864
C27	0.3114	0.14474	0.56711
N28	0.35038	0.19127	0.50241
C29	0.22701	0.14782	0.45351
C30	0.16789	0.11928	0.41269
C31	0.12905	0.0606	0.45199

C32	0.14876	0.03289	0.54135
C33	0.20774	0.06145	0.5833
C34	0.24782	0.11844	0.53631
O35	0.07185	0.02833	0.39927
N36	0.04287	0.06803	0.16413
C37	0.63634	0.36551	0.91011
C38	0.69845	0.39512	0.91353
C39	0.82986	0.37188	0.91465
C40	0.88571	0.39308	0.85651
C41	0.90639	0.43765	0.75902
C42	0.87122	0.46237	0.72381
C43	0.81569	0.44127	0.78147
C44	0.79317	0.39431	0.87406
C45	0.99211	0.502	0.62359
N46	0.96052	0.4532	0.68987
C47	0.05686	0.46654	0.52164
C48	0.10928	0.47865	0.45709
C49	0.15166	0.5369	0.4246
C50	0.13809	0.58218	0.44356
C51	0.08553	0.57013	0.50661
C52	0.04545	0.51266	0.55012
O53	0.2065	0.54791	0.37564
P54	0.26854	0.60627	0.45701
C55	0.03015	0.96733	0.80907
C56	0.96786	0.93813	0.80986
C57	0.84112	0.96157	0.7284
C58	0.7795	0.92954	0.71244
C59	0.74563	0.87301	0.76831
C60	0.77481	0.84981	0.84469
C61	0.83642	0.88175	0.86
C62	0.87079	0.93788	0.80112
C63	0.64633	0.78529	0.76922
N64	0.68246	0.84115	0.74483
C65	0.55563	0.7878	0.6864
C66	0.49508	0.75802	0.65432
C67	0.46023	0.69659	0.67381
C68	0.48615	0.66594	0.72983

C69	0.54664	0.69527	0.75952
C70	0.58214	0.75641	0.73825
O71	0.40076	0.66496	0.63386
N72	0.39508	0.72403	0.40268
H73	0.43645	0.60242	-0.00696
H74	0.5349	0.618	0.03624
H75	0.58646	0.78864	0.20691
H76	0.48824	0.77187	0.17927
H77	0.64574	0.76258	0.31569
H78	0.72305	0.70365	0.09165
H79	0.83091	0.75481	0.05764
H80	0.84835	0.89647	0.31459
H81	0.74411	0.84462	0.35277
H82	0.53371	0.33682	0.37068
H83	0.42566	0.28825	0.37406
H84	0.41918	0.15685	0.64778
H85	0.52619	0.20588	0.647
H86	0.32465	0.12224	0.63806
H87	0.25666	0.1922	0.41757
H88	0.15281	0.14201	0.34675
H89	0.11922	-0.01233	0.57109
H90	0.22262	0.03797	0.64751
H91	0.81457	0.3364	0.9873
H92	0.91225	0.3735	0.8846
H93	0.88458	0.49558	0.64625
H94	0.789	0.45935	0.74578
H95	0.98102	0.53679	0.62481
H96	0.02591	0.42147	0.55119
H97	0.11863	0.44319	0.43975
H98	0.1678	0.6268	0.41066
H99	0.07674	0.60602	0.52379
H100	0.86547	1.00415	0.68002
H101	0.75812	0.94849	0.65385
H102	0.75079	0.80723	0.89313
H103	0.8572	0.8621	0.91734
H104	0.66273	0.75762	0.80864
H105	0.58168	0.83502	0.66922

H106	0.47546	0.78259	0.6132
H107	0.45985	0.61858	0.74462
H108	0.56595	0.66983	0.79752
H109	0.41858	0.76842	0.06076
H110	0.60681	0.22375	0.50841
H111	0.7231	0.44286	0.90708
H112	0.94317	0.89034	0.80865

Table S5 Unit cell parameters and fractional atomic coordinates for JUC-612 calculated based on the eclipsed ***hcb*** net

Space group		*P*3	
Calculated unit cell		$a = b = 29.3969$ Å, $c = 3.5123$ Å, $\alpha = \beta = 90°$, $\gamma = 120°$	
Measured unit cell		$a = b = 28.9889$ Å, $c = 3.5156$ Å, $\alpha = \beta = 90°$, $\gamma = 120°$	
Pawley refinement		*R*p = 2.07%, *R*wp = 2.68%	
atoms	x	y	z
C1	-0.33409	-0.2859	0.45758
C2	-0.28608	-0.24086	0.48048
C3	-0.24031	-0.24301	0.52607
C4	-0.24365	-0.29194	0.54935
C5	-0.29235	-0.33737	0.52788
C6	-0.33686	-0.33385	0.48233
C7	-0.1427	-0.19298	0.56559
C8	-0.09593	-0.1457	0.57968
C9	-0.09576	-0.09842	0.57695
C10	-0.14323	-0.09946	0.56126
C11	-0.18967	-0.14642	0.54682
C12	-0.19013	-0.19376	0.54785
C13	-0.04664	-0.04805	0.58477
C14	0.38005	0.66886	0.27104
C15	0.43276	0.62538	0.29488
C16	0.48041	0.62737	0.32286
C17	0.52627	0.67507	0.34316
C18	0.52421	0.72109	0.33232
C19	0.4765	0.71907	0.30414
C20	0.43045	0.67118	0.28645

C21	0.61881	0.71731	0.41139
N22	0.57413	0.67544	0.38307
N23	0.86275	1.26456	0.5891
C24	0.9107	1.30069	0.55322
C25	0.83538	1.33042	0.61354
C26	0.79665	1.34356	0.62889
C27	0.74447	1.3049	0.64229
C28	0.73169	1.25267	0.64195
C29	0.77069	1.23941	0.62694
C30	0.82286	1.27821	0.61133
C31	0.70396	1.31958	0.65049
N32	0.37765	0.71316	0.27157
N33	0.7174	0.37044	0.6502
C34	1.04611	1.04743	0.58454
H35	-0.28339	-0.20139	0.46229
H36	-0.20696	-0.29449	0.58538
H37	-0.37603	-0.37101	0.4653
H38	-0.14232	-0.23105	0.56852
H39	-0.05752	-0.14533	0.59352
H40	-0.1441	-0.06165	0.56023
H41	-0.22809	-0.1468	0.5339
H42	0.39543	0.58634	0.27874
H43	0.48203	0.58996	0.32924
H44	0.56152	0.76016	0.34653
H45	0.47488	0.75648	0.29542
H46	0.62152	0.75683	0.40035
H47	0.92107	1.34303	0.53902
H48	0.87784	1.36216	0.60256
H49	0.8074	1.38604	0.63059
H50	0.68935	1.22067	0.65395
H51	0.75993	1.19693	0.62741
H52	1.08355	1.08645	0.58418

Table S6 Unit cell parameters and fractional atomic coordinates for JUC-612 calculated based on the staggered ***hcb*** net

Space group	*P*3

Calculated unit cell		$a = b = 29.9375$ Å, $c = 7.1565$ Å, $\alpha = \beta = 90°$, $\gamma = 120°$	
atoms	x	y	z
C1	0.66819	0.71824	0.22929
C2	0.71649	0.76154	0.26155
C3	0.76009	0.75665	0.29932
C4	0.75447	0.70681	0.29936
C5	0.7061	0.66323	0.26135
C6	0.66348	0.66941	0.2282
C7	0.85716	0.80856	0.26685
C8	0.90358	0.85565	0.2779
C9	0.90527	0.89968	0.35724
C10	0.85926	0.89411	0.43173
C11	0.81302	0.84741	0.41876
C12	0.81092	0.80423	0.33225
C13	0.95403	0.95122	0.35964
C14	0.38059	0.67043	0.10548
C15	0.43612	0.63003	0.12967
C16	0.48444	0.63411	0.14672
C17	0.52935	0.68243	0.14814
C18	0.52543	0.72705	0.12594
C19	0.47707	0.72301	0.10877
C20	0.43186	0.67451	0.11293
C21	0.62283	0.72434	0.19773
N22	0.57755	0.68391	0.17557
N23	0.86972	0.2748	0.33671
C24	0.91303	0.30276	0.24915
C25	0.83878	0.33802	0.31075
C26	0.79857	0.3488	0.30933
C27	0.74769	0.30974	0.34364
C28	0.73803	0.25949	0.38099
C29	0.77845	0.24856	0.38056
C30	0.82904	0.28737	0.34159
C31	0.70544	0.32194	0.34175
N32	0.37652	0.71359	0.10576
N33	0.71644	0.37184	0.34172
C34	0.04524	0.04794	0.35949

H35	0.72001	0.79929	0.25437
H36	0.78727	0.70189	0.3276
H37	0.62624	0.63565	0.20103
H38	0.85713	0.77647	0.19717
H39	0.93739	0.85775	0.215
H40	0.8586	0.92555	0.50344
H41	0.77878	0.84514	0.479
H42	0.40215	0.59202	0.13258
H43	0.48691	0.59943	0.16238
H44	0.55907	0.76508	0.12259
H45	0.47516	0.75802	0.0942
H46	0.62801	0.76273	0.1989
H47	0.91922	0.33605	0.16945
H48	0.8774	0.36994	0.29017
H49	0.80757	0.38802	0.2822
H50	0.69928	0.22862	0.41053
H51	0.77023	0.20962	0.40817
H52	0.08021	0.08487	0.35812
C53	0.33352	0.04871	0.76208
C54	0.38239	0.09241	0.76076
C55	0.42737	0.08852	0.76843
C56	0.42208	0.03876	0.75963
C57	0.37255	0.99447	0.75195
C58	0.32887	1	0.7563
C59	0.52464	0.14139	0.7246
C60	0.57106	0.18866	0.73495
C61	0.57271	0.23256	0.81535
C62	0.52692	0.22682	0.89145
C63	0.48097	0.18003	0.88092
C64	0.47847	0.13679	0.79272
C65	0.62108	0.28431	0.81737
C66	0.04718	0.00378	0.86429
C67	0.10385	0.96531	0.90228
C68	0.1519	0.96871	0.8875
C69	0.19497	0.01438	0.82695
C70	0.18944	0.05713	0.78163
C71	0.14149	0.05387	0.79763

C72	0.09819	0.00783	0.85649
C73	0.28776	0.05465	0.77586
N74	0.24276	0.01474	0.8072
N75	0.53273	0.60381	0.68291
C76	0.57806	0.6369	0.74707
C77	0.50387	0.66742	0.62217
C78	0.46452	0.67926	0.60659
C79	0.41265	0.64052	0.6233
C80	0.4011	0.58926	0.65148
C81	0.44073	0.57743	0.66806
C82	0.49249	0.61649	0.65839
C83	0.37128	0.65405	0.6156
N84	0.04317	0.04694	0.86403
N85	0.38347	0.70433	0.61589
C86	0.71159	0.38153	0.81707
H87	0.38488	0.12971	0.75628
H88	0.45567	0.03418	0.76585
H89	0.29084	0.96592	0.75458
H90	0.52465	0.10944	0.65402
H91	0.60485	0.19106	0.67047
H92	0.52623	0.25818	0.96355
H93	0.44743	0.17804	0.94581
H94	0.07118	0.92942	0.94913
H95	0.15562	0.93552	0.92221
H96	0.22124	0.09269	0.72965
H97	0.13808	0.08702	0.75903
H98	0.29296	0.09301	0.76797
H99	0.58594	0.6748	0.79049
H100	0.54315	0.69833	0.60327
H101	0.47489	0.71894	0.58094
H102	0.3615	0.55838	0.66411
H103	0.43111	0.53793	0.6928
H104	0.74636	0.41864	0.81467

Table S7 Comparison of scalability and material quality of nanosheets fabricated via exfoliation of pre-synthesized COFs

Materials	Thickness (nm)	Lateral Size	Refs.
**JUC-610-CONs**	~4	~800 nm	**This work**
DAAQ-ECOF	5	~400 nm	[S4]
EB-TFP-iCONs	~1.5	~500 nm	[S5]
TTA-DFP CONs	1.1	~200 nm	[S6]
Ag+@TpHz Nanosheets	4.6	~600 nm	[S7]
COF-8 Nanosheets	4	~2 μm	[S8]
Ketoenamine-linked CONs	3-10	~2 μm	[S9]
Hydrazone-linked CONs	~1.32	~200 nm	[S10]
2D Conjugated Aromatic Polymer Nanosheets	~1.0	15 μm	[S11]
π-conjugated Porous Organic Nanosheets	2-5	1-10 μm	[S12]
iCONs	2-5	~500 nm	[S13]
CMP0 Nanosheets	3.4	~2 μm	[S14]
π-conjugated CONs	~1.4	~400 nm	[S15]
π-conjugated CONs	3.1	2-20 μm	[S16]
TPA-COF Nanosheets	~3.5	~300 nm	[S17]
PI-CONs	~1.2	5~6 μm	[S18]

Table S8 Metal-free organic porous materials without pyrolysis of ORR reactions

Materials	Half-wave/V Potential / V	Oneset Potential/V	Ref.
**JUC-610-CONs**	0.72	0.83	**This work**
H-TP-COF	0.65	0.71	[S19]
JUC-528	0.7	0.82	[S20]
TZA-COF	0.75	0.8	[S21]
TAPTt	0.74	0.85	[S22]
PTCOF	0.7	0.8	[S23]
PTM-H-COF@C	0.62	0.75	[S24]
PTM-CORF@C	0.7	0.78	[S24]

TAPA-NDI-Super P	0.71	0.92	[S25]
DAPT-TFP-COF / GC	0.67	0.8	[S26]
COF-JLU-82	0.68	0.98	[S27]
COF-JLU-23	0.66	0.99	[S27]
DAF-COF	0.74	0.89	[S28]
Azo-COF	0.68	0.88	[S29]
HHPC	0.78	0.8	[S30]
NCNs	0.82	0.81	[S31]
DN-UGNR	0.753	1.072	[S32]
NSCHCT	0.84	0.85	[S33]

Table S9 The ORR performance of selected metal-based catalysts

Catalyst	Half-wave/V Potential / V	E/V Δ	Ref.
LDH-POF	0.8	0.68	[S34]
S-CFZ	0.82	0.7	[S35]
CoDNG-900	0.864	0.749	[S36]
CMO–U@CC	0.81	0.672	[S37]
NiFe/bNCNT	0.8	0.76	[S38]

Supplementary References

[S1] T.-M. Geng, F.-Q. Wang, X.-C. Fang, H. Xu, Dual functional N,O,P containing covalent organic frameworks for adsorping iodine and fluorescence sensing to p-nitrophenol and iodine. Microporous Mesoporous Mater. **317,** 111001 (2021). https://doi.org/10.1016/j.micromeso.2021.111001

[S2] W. Meng, J.K. Clegg, J.D. Thoburn, J.R. Nitschke, Controlling the transmission of stereochemical information through space in terphenyl-edged Fe4L6 cages. J. Am. Chem. Soc. **133,** 13652 (2021). https://doi.org/10.1021/ja205254s

[S3] A.F.M. El-Mahdy, C.-H. Kuo, A. Alshehri, C. Young, Y. Yamauchi et al., Strategic design of triphenylamine- and triphenyltriazine-based two-dimensional covalent organic frameworks for CO_2 uptake and energy storage. J. Mater. Chem. A **6,** 19532 (2018). https://doi.org/10.1039/C8TA04781B

[S4] S. Wang, Q. Wang, P. Shao, Y. Han, X. Gao et al., Exfoliation of covalent organic frameworks into few-layer redox-active nanosheets as cathode materials for lithium-ion batteries. J. Am. Chem. Soc. **139,** 4258 (2017). https://doi.org/10.1021/jacs.7b02648

[S5] A. Mal, R.K. Mishra, V.K. Praveen, M.A. Khayum, R. Banerjee et al., Supramolecular reassembly of self-exfoliated ionic covalent organic nanosheets for label-free detection of double-stranded DNA. Angew. Chem. Int. Ed. **57,** 8443 (2018). https://doi.org/10.1002/anie.201801352

[S6] S. Chandra, S. Kandambeth, B.P. Biswal, B. Lukose, S.M. Kunjir et al., Chemically stable multilayered covalent organic nanosheets from covalent organic frameworks via mechanical delamination. J. Am. Chem. Soc. **135,** 17853 (2013). https://doi.org/10.1021/ja408121p

[S7] M. Wang, F. Pan, H. Yang, Y. Cao, H. Wang et al., Constructing channel-mediated facilitated transport membranes by incorporating covalent organic framework nanosheets with tunable microenvironments. J. Mater. Chem. A **7,** 9912 (2019). https://doi.org/10.1039/C8TA11883C

[S8] I. Berlanga, M. Luisa Ruiz-Gonzalez, J. Maria Gonzalez-Calbet, J.L.G. Fierro, R. Mas-Balleste et al., Delamination of layered covalent organic frameworks. Small **7,** 1207 (2011). https://doi.org/10.1002/smll.201002264

[S9] D.N. Bunck, W.R. Dichtel, Bulk synthesis of exfoliated two-dimensional polymers using hydrazone-linked covalent organic frameworks. J. Am. Chem. Soc. **135,** 14952 (2013). https://doi.org/10.1021/ja408243n

[S10] W. Liu, X. Luo, Y. Bao, Y.P. Liu, G.-H. Ning et al., A two-dimensional conjugated aromatic polymer via C–C coupling reaction. Nat. Chem. **9,** 563 (2017). https://doi.org/10.1038/nchem.2696

[S11] J. Dong, K. Zhang, X. Li, Y. Qian, H. Zhu et al., Ultrathin two-dimensional porous organic nanosheets with molecular rotors for chemical sensing. Nat. Comm. **8,** 1142 (2017). https://doi.org/10.1038/s41467-017-01293-x

[S12] S. Mitra, S. Kandambeth, B.P. Biswal, A.M. Khayum, C.K. Choudhury et al., Self-exfoliated guanidinium-based ionic covalent organic nanosheets (iCONs). J. Am. Chem. Soc. **138,** 2823 (2016). https://doi.org/10.1021/jacs.5b13533

[S13] S. Chandra, S. Kandambeth, B.P. Biswal, B. Lukose, S.M. Kunjir et al., Chemically stable multilayered covalent organic nanosheets from covalent organic frameworks via mechanical delamination. J. Am. Chem. Soc. **135,** 17853 (2013). https://doi.org/10.1021/ja408121p

[S14] I. Berlanga, R. Mas-Balleste, F. Zamora, Tuning delamination of layered covalent organic frameworks through structural design. Chem. Comm. **48,** 7976 (2012). https://doi.org/10.1039/C2CC32187D

[S15] M.R. Rao, Y. Fang, S. De Feyter, D.F. Perepichka, Conjugated covalent organic frameworks via michael addition–elimination. J. Am. Chem. Soc. **139,** 2421 (2017). https://doi.org/10.1021/jacs.6b12005

[S16] J. Dong, X. Li, K. Zhang, Y. Di Yuan, Y. Wang et al., Confinement of aggregation-induced emission molecular rotors in ultrathin two-dimensional porous organic nanosheets for enhanced molecular recognition. J. Am. Chem. Soc. **140,** 4035 (2018). https://doi.org/10.1021/jacs.7b13069

[S17] Y. Peng, Y. Huang, Y. Zhu, B. Chen, L. Wang et al., Ultrathin two-dimensional covalent organic framework nanosheets: preparation and application in highly sensitive and selective DNA detection. J. Am. Chem. Soc. **139,** 8698 (2017). https://doi.org/10.1021/jacs.7b04096

[S18] H. Duan, K. Li, M. Xie, J.-M. Chen, H.-G. Zhou et al., Scalable synthesis of ultrathin polyimide covalent organic framework nanosheets for high-performance lithium–sulfur batteries. J. Am. Chem. Soc. **143,** 19446 (2021). https://doi.org/10.1021/jacs.1c08675

[S19] J.-Y. Yue, Y.-T. Wang, X. Wu, P. Yang, Y. Ma et al., Two-dimensional porphyrin covalent organic frameworks with tunable catalytic active sites for the oxygen reduction reaction. Chem. Comm. **57,** 12619 (2021). https://doi.org/10.1039/D1CC04928C

[S20] D. Li, C. Li, L. Zhang, H. Li, L. Zhu et al., Metal-free thiophene-sulfur covalent organic frameworks: precise and controllable synthesis of catalytic active sites for oxygen reduction. J. Am. Chem. Soc. **142,** 8104 (2020). https://doi.org/10.1021/jacs.0c02225

[S21] S. Roy, S. Mari, M.K. Sai, S.C. Sarma, S. Sarka et al., Highly efficient bifunctional oxygen reduction/evolution activity of a non-precious nanocomposite derived from a tetrazine-COF. Nanoscale **12,** 22718 (2020). https://doi.org/10.1039/D0NR05337F

[S22] C. Liu, F. Liu, H. Li, J. Chen, J. Fei et al., One-dimensional van der waals heterostructures as efficient metal-free oxygen electrocatalysts. Acs Nano **15,** 3309 (2021). https://doi.org/10.1021/acsnano.0c10242

[S23] J.H. Park, C.H. Lee, J.-M. Ju, J.-H. Lee, J. Seol et al., Bifunctional covalent organic framework-derived electrocatalysts with modulated p-band centers for rechargeable Zn–air batteries. Adv. Funct. Mater. **31,** 2101727 (2021). https://doi.org/10.1002/adfm.202101727

[S24] S. Wu, M. Li, H. Phon, D. Wang, T. Herng et al., Toward two-dimensional π-conjugated covalent organic radical frameworks. Angew. Chem. Int. Ed. **57,** 8007 (2018). https://doi.org/10.1002/anie.201801998

[S25] M. Martinez-Fernandez, E. Martinez-Perinan, S. Royuela, J.I. Martinez, F. Zamora et al., Covalent organic frameworks based on electroactive naphthalenediimide as active electrocatalysts toward oxygen reduction reaction.

Appl. Mater. Today. **26,** 101384 (2022).https://doi.org/10.1016/j.apmt.2022.101384

[S26] P. Garcia-Arroyo, E. Martinez-Perinan, J.J. Cabrera-Trujillo, E. Salagre, E.G. Michel et al., Pyrenetetraone-based covalent organic framework as an effective electrocatalyst for oxygen reduction reaction. Nano Res. **15,** 3907 (2022). https://doi.org/10.1007/s12274-021-4043-2

[S27] J. Jia, J. Li, S. Ma, Z. Zhang, X. Liu, Metal-free covalent organic frameworks for electrocatalytic oxygen reduction reaction. Macromol. Rapid. Comm. 2200717 (2022). https://doi.org/10.1002/marc.202200717

[S28] Z. You, B. Wang, Z. Zhao, Q. Zhang, W. Song et al., Metal-free carbon-based covalent organic frameworks with heteroatom-free units boost efficient oxygen reduction. Adv. Mater. **35,** 2209129 (2023). https://doi.org/10.1002/adma.202209129

[S29] X. Yan, B. Wang, J. Ren, X. Long, D. Yang, An unsaturated bond strategy to regulate active centers of metal-free covalent organic frameworks for efficient oxygen reduction. Angew. Chem. Int. Ed. **61,** 3 (2022). https://doi.org/10.1002/anie.202209583

[S30] X. Xiao, X. Li, Z. Wang, G. Yan, H. Guo et al., Robust template-activator cooperated pyrolysis enabling hierarchically porous honeycombed defective carbon as highly-efficient metal-free bifunctional electrocatalyst for Zn-air batteries. Appl. Catal. B-Environ. **265,** 118603 (2020). https://doi.org/10.1016/j.apcatb.2020.118603

[S31] H. Jiang, J. Gu, X. Zheng, M. Liu, X. Qiu et al., Defect-rich and ultrathin N doped carbon nanosheets as advanced trifunctional metal-free electrocatalysts for the ORR, OER and HER. Energy Environ. Sci. **12,** 322 (2019). https://doi.org/10.1039/C8EE03276A

[S32] J. Zhang, Y. Sun, J. Zhu, Z. Gao, S. Li et al., Ultranarrow graphene nanoribbons toward oxygen reduction and evolution reactions. Adv. Sci. **5,** 1801375 (2018). https://doi.org/10.1002/advs.201801375

[S33] Z. Li, Y. Yao, Y. Niu, W. Zhang, B. Chen et al., Multi-heteroatom-doped hollow carbon tubes as robust electrocatalysts for the oxygen reduction reaction, oxygen and hydrogen evolution reaction. Chem. Eng. J. **418,** 129321 (2021). https://doi.org/10.1016/j.cej.2021.129321

[S34] C. Zhao, J. Liu, B. Li, D. Ren, X. Chen et al., Multiscale construction of bifunctional electrocatalysts for long-lifespan rechargeable zinc–air batteries. Adv. Funct. Mater. **30,** 2003619 (2020). https://doi.org/10.1002/adfm.202003619

[S35] Y. Jiang, Y. Deng, R. Liang, N. Chen, G. King et al., Linker-compensated metal–organic framework with electron delocalized metal sites for bifunctional oxygen electro catalysis. J. Am. Chem. Soc. **144,** 4783 (2022). https://doi.org/10.1021/jacs.1c10295

[S36] A. Wang, C. Zhao, M. Yu, W. Wang, Trifunctional Co nanoparticle confined in defect-rich nitrogen-doped graphene for rechargeable Zn-air battery with a long lifetime. Appl. Catal. B. Environ. **281,** 119514 (2021). https://doi.org/10.1016/j.apcatb.2020.119514

[S37] G. Janani, S. Surendran, H. Choi, M. Han, U. Sim, In situ grown $CoMn_2O_4$ 3D-tetragons on carbon cloth: flexible electrodes for efficient rechargeable zinc–air battery powered water splitting systems. Small **17,** 2103613 (2021). https://doi.org/ 10.1002/smll.202103613

[S38] J. Hong, G. Park, Y. Kang, Aerosol-assisted synthesis of bimetallic nanoparticle-loaded bamboo-like N-doped carbon nanotubes as an efficient bifunctional oxygen catalyst for Zn-air batteries. Int. J. Energy. Res. **46,** 5215 (2022). https://// doi.org/10.1002/er.7513
